# Supplementary material for: A qualitative study to examine hidden care burden for older adults with overweight and obesity in England
Source: PLoS One. 2025 Mar 19;20(3):e0320253. doi: 10.1371/journal.pone.0320253 (PMC11922259; doi:10.1371/journal.pone.0320253)
Supplement: S1 File — (DOCX) [file pone.0320253.s001.docx]

Consent Form (4/12/21, Version- 0.2)

IRAS ID: 253586

Centre Name: Addison House Surgery, Harlow

Study Number:

Participant Identification Number for this trial:

**CONSENT FORM**

Title of Project: The impact of obesity on health and social care needs among older adults (50+) in England

Name of Principal investigator: Professor Hafiz Khan

Name of Doctoral research student: Mrs Gargi Ghosh

Please initial box

1. I confirm that I have read the information sheet dated.................... (Version 0.2) for the above study. I have had the opportunity to consider the information, ask questions and have had these answered satisfactorily.
2. I understand that my participation is voluntary and that I am free to withdraw at any time without giving any reason, without my medical care or legal rights being affected.
3. I understand that the data collected during the study (without any of my identifiable information), may be looked at by individuals from [University of West London], from regulatory authorities or from the NHS Trust, where it is relevant to my taking part in this research. I give permission for these individuals to have access to my records.
4. I understand that the information collected about me will be used to support other research in the future and may be shared anonymously with other researchers.
5. I consent to use my interview transcripts, with all identifiable details removed, for teaching, future research, and publication purposes.
6. I agree to my General Practitioner being informed of my participation in the study.
7. I understand that the information held and maintained by the research team at the University of West London is a password-protected server.
8. I agree to take part in the above study.

Name of Participant Date Signature

Name of Person taking consent Date Signature
