# Supplementary material for: A qualitative study to examine hidden care burden for older adults with overweight and obesity in England
Source: PLoS One. 2025 Mar 19;20(3):e0320253. doi: 10.1371/journal.pone.0320253 (PMC11922259; doi:10.1371/journal.pone.0320253)
Supplement: S2 File — (DOCX) [file pone.0320253.s002.docx]

Data Management and Storage Statement

All applicants should familiarise themselves with the Data Protection Act 1998, and adhere to its principles in all aspects of their research:

<http://www.uwl.ac.uk/sites/default/files/Departments/About-us/Web/PDF/policies/policy-on-data-protection.pdf>

As a student or member of staff undertaking a research project, I understand that I am responsible for the following:

- The security and confidentiality of all data collected.
- Mitigating all risks to anonymity, privacy and confidentiality posed by all kinds of personal information storage, processing, including computer and paper files, e-mail records, audio and video files, and any information that directly identifies an individual.

I will ensure that:

- Data and codes and all identifying information will be kept in separate locked filing cabinets/files and working files will contain no identifying information and will only be accessed by one or two persons.
- All recordings will be transcribed using codes or pseudonyms for identification of individuals and destroyed upon completion of the research project.
- Access to computer files will be available by password only.
- Data will be stored for up to 5 years after the end of the project, after which they should be disposed of safely.

Name: Gargi Ghosh Application ID: 21374279

**Project title:** The impact of obesity on health and social care needs among older adults (50+) in England.
