# Supplementary material for: A qualitative study to examine hidden care burden for older adults with overweight and obesity in England
Source: PLoS One. 2025 Mar 19;20(3):e0320253. doi: 10.1371/journal.pone.0320253 (PMC11922259; doi:10.1371/journal.pone.0320253)
Supplement: S4 File — (DOCX) [file pone.0320253.s004.docx]

GP Information Sheet (22/05/18, Version 0.1)

**Study title** – The impact of obesity on health and social care needs among older adults (50+) in England.

Name of Principal investigator (PI) & Academic supervisor: **Professor Hafiz T.A Khan**

Co-supervisor: **Dr Salim Vohra**

Doctoral research student: **Mrs Gargi Ghosh**

I am a doctoral research student. I have started my PhD in Public health in January 2018 under University of West London. I am undertaking this above study as a part of my PhD project. It is a 3years project and will end roughly in December 2020.

**What is the purpose of the study?** Today there are one quarter obese adults in England. This highlights the need to promote effective health and social care to improve wellbeing and quality-of-life for adults with increased weight. and at the same time the older adults with or without obesity have the right to live a well-supported and honourable life at the very end of their life journey and as a health professional and as a researcher it is my responsibility to find out the factors that would promote their health and wellbeing.

In addition, by doing this study, both medical, nursing staff and health policy makers would be beneficial as they can obtain the latest knowledge in regard to this topic. Finally, this study will also provide a means of overall cost effectiveness.

**Aim and objectives**: The primary aim of the study is to explore the effect of obesity on health and social care needs among older adults in England.

Objectives:

The specific research objectives are as follows:

i. To investigate the association between obesity, disability status, comorbidity in older adults.

ii. To determine the association between current health status and wellbeing in older adults with obesity.

iii. To explore the differences in social care received by degree of obesity.

iv. To examine the role of obesity in determining unmet social care needs in older adults.

**Research design and planning**: The study would follow a mixed-methods design by using both quantitative data (from the English Longitudinal Study on Ageing (ELSA) and qualitative data to explore the research questions and satisfy the research objectives. Although quantitative data would be thoroughly analysed to answer some of my research questions, however quantitative data is not enough to answer all my research questions. Therefore, to understand the social picture, qualitative interview will follow. In addition, qualitative interview will help me to cross check some of the findings of quantitative research and also testing some of my research questions.

Therefore, for this study, qualitative interviews will be undertaken on a sample of 30-40 older adults (50+), in which each obese (BMI ≥30) and non-obese (BMI≤ 30) group would have15-20 older adults (I will check their height and weight and calculate their BMI on the spot, so I can divide the participants in two groups- obese and non- obese). They will all be given a semi-structured questionnaire for face to face and one to one real time conversation or ‘guided conversation’ through their responses to the questionnaire. The responses will be documented in situ by the researcher on individual questionnaires.

An in-depth one to one interview would follow at the same time with a sample of 10 (will be drawn randomly from the previously mentioned sample of face-to face interviewees), 5 from each group would be randomly selected for interview having previously been grouped on the basis of their BMI (I will check their height and weight and calculate their BMI on the spot).

These participants would be randomly selected from the Patients list of the minor illness clinic of an NHS GP surgery.

The recruited population groups have to satisfy the inclusion and exclusion criteria, set out for this study listed below:

Inclusion criteria:

Population group to be included in this study should satisfy following criteria-

- Older adults of 50 years and over
- Can speak and understand English.
- Older adults visiting to minor illness clinic.
- Older adults BMI ≥18.5 kg/m2

Exclusion criteria:

- Patients with a history of any of the following will be excluded-
- Cannot speak and understand English.
- Advanced stage dementia
- Individual with severe or profound ID (intellectual disabilities), individual with Prader Willi syndrome, Cohen syndrome or Bardet-Biedl syndrome

**Ethical Considerations**: Every participant has to give written consent before participating in the study. Every participant would be explained the purpose, design of the study and involvement to potential recruits by me as an interviewer. The participants will also be reassured that the care that they would normally receive will not be affected by their decision to participate or not and that they can withdraw the consent at any point. Their personal identity will be protected at all times; all data will be entered in the study only by case number. An IRAS (The Integrated Research Application System) application form will be filled in to obtain permission and ethical body approval for NHS patients. The University ethical body approval application is already submitted.

**Expected Contribution**: The project aims to contribute to the field through:

- Providing an update to the obesity care pathway toolkit, developed by the National Obesity Forum which is too generalised and lacks the detail needed to identify the complex care needs of obese older adults.
- Providing research outputs to inform others in the field who are also designing services for older people and contributing to this body of knowledge.
- Contributing to social care policy.

**Dissemination of Findings**: The results of this study will form a major component of my final thesis. Depending on the findings, the study results may be presented at Doctoral conferences, Workshop, Seminar, Publications, Etc. No identifiable participant’s information will be included in any of the reports. However, having completed the research I will share the executive summary of the study with the participants and their GPs.

**How long does the study last?** The actual study would take place between June 2018 and December 2020. As mentioned above, Participant’s involvement should only last about 30-40 minutes.

**What are the potential disadvantages and risks of taking part?** There are no significant risks associated with taking part. As the participant’s confidentiality will always be protected and I am going to denote each participant as a case no. in this research project. However, I will take the participant’s name and email address/ postal address, so that I can share the study findings with the participants and their GPs after completion and these will be saved in a password protected file of university computer, which only I and chief investigator can access.

**What are the possible benefits of taking part?** As this study does not involve treatment, there are no direct benefits of taking part. The study, however, will provide useful insights into patients’ perspectives. It will also increase our understanding of the relationship between patients’ attitudes and expectation towards the health and social care received. Such information has the potential to benefit patient care in future.

**What if there is a problem? Complaints:** If you have a concern about any aspect of this study, you can speak to me directly or email me (21374279@student.uwl.ac.uk) and I will do my best to answer your questions, or you can directly contact my academic supervisor / PI: hafiz.khan@uwl.ac.uk.

**Further information and contact details:**

For specific information about this project feel free to contact me – E-mail: 21374279@student.uwl.ac.uk OR my academic supervisor / PI: hafiz.khan@uwl.ac.uk.

Last Modified 1st October 2019
