# Supplementary material for: A qualitative study to examine hidden care burden for older adults with overweight and obesity in England
Source: PLoS One. 2025 Mar 19;20(3):e0320253. doi: 10.1371/journal.pone.0320253 (PMC11922259; doi:10.1371/journal.pone.0320253)
Supplement: S5 File — (DOCX) [file pone.0320253.s005.docx]

Participants’ Information Sheet

**Participant Information Sheet (04/12/19, Version 0.3)**

**Study title** – The impact of obesity on health and social care needs among older adults (50+) in England.

IRAS ID: 253586

Centre Name: Addison House Surgery, Harlow

Doctoral research student: Mrs Gargi Ghosh

I am a doctoral research student, and I would like to invite you to take part in my research project which takes the form of a survey. Before you decide, I would like you to understand why the study is being done and what it would mean for you. I will go through this information sheet with you and answer any questions you may have. I’d suggest this should take about 5 minutes.

In this research study we will use information from you. We will only use information that we need for the research study. We will let very few people know your name, and only if they really need it for this study.

Everyone involved in this study will keep your data safe and secure. We will also follow all privacy rules.

At the end of the study, we will save some of the data in case we need to check it.

We will make sure no-one can work out who you are from the reports we write.

The information pack tells you more about this.

**How will we use information about you?**

We will need to use information from you for this research project.

This information will include your name.

People who do not need to know who you are will not be able to see your name. Your data will have a code number instead. I will only ask about your existing care and support and if that is making a difference to your health, wellbeing and quality of life.

We will keep all information about you safe and secure.

Once we have finished the study, we will keep some of the data so we can check the results. We will write our reports in a way that no-one can work out that you took part in the study.

**What are your choices about how your information is used?**

You can stop being part of the study at any time, without giving a reason, but we will keep information about you that we already have.

We need to manage your records in specific ways for the research to be reliable. This means that we won’t be able to let you see or change the data we hold about you.

**Where can you find out more about how your information is used?**

You can find out more about how we use your information.

at www.hra.nhs.uk/information-about-patients/

our leaflet available from: www.hra.nhs.uk/patientdataandresearch

by asking one of the research team

by sending an email to: 21374279@student.uwl.ac.uk, or

by ringing us on -02082312953.

**What is the purpose of the study?**

Today there are one quarter obese adults in England. With increasing life expectancy, the population is ageing alongside this increase in obesity. Obesity coupled with the challenges of ageing, leads to an unfortunate burden of chronic diseases for family and carers. Specially, obesity in older adults is more complex than young population, due to the presence of degenerative muscle loss. The existing national guidelines for the care and support needs for the people with obesity, fails to address the care pathway for older adults with obesity for all aspects of their wellbeing and quality of life related to their current health status.

Moreover, the Health survey for England established that there is clear evidence of need for care and support for older adults with obesity, but this survey does not report on any particular types of care needs for obese older adults.

This highlights the need to find out the factors that would promote effective health and social care to improve wellbeing and quality of life for older adults with increased weight.

In addition, by doing this study, both medical, nursing staff and health policy makers would be beneficial as they can obtain the latest knowledge in regard to this topic. It will also increase our understanding of the relationship between patients’ attitudes and expectation towards the health and social care received. Such information has the potential to benefit patient care in future. Finally, this study will also provide a means of overall cost effectiveness.

**Why does this study use information from patients?**

This purpose of the study is to find out the effect of obesity on health and social care needs among older adults in England, to explore the differences in social care received by degree of obesity. Finally, to examine the role of obesity in determining social care needs in older adults. The information I will collect from you will show how satisfied you are at your home with the existing care and support and if it is making a difference to your health, wellbeing and quality of life. For this purpose, you have to sign a consent form. But researchers must always make sure that as few people as possible can see this sort of information that can show who you are.

This study will only use your data that really needs to do the research. Most of the research team will not need to know your name. In these cases, I will remove your name from the research data and replace it with a code number. This is called coded data, or the technical term is pseudonymised data. It can be matched up with the rest of the data relating to you by the code number. No identifiable information would be collected from you for this study. You will be identified with a numerical case number, so your confidentiality will be protected all the time.

**Why have I been invited?**

You were invited to take part in this study because you fulfil the criteria used to select individuals for this study. The criteria are as follows:

Older adults of 50 years and over

Can speak and understand English.

**Do I have to take part?**

The decision to take part in this study is completely up to you. I will describe the study and go through this information sheet with you. If you agree to take part, you will be required to sign a consent form. If you need more time to think about it, you can take this information sheet and the consent form with you and contact me (my email id is: 21374279@student.uwl.ac.uk) within 2 weeks from now, if you want to take part for this study. You are free to withdraw at any time, without giving a reason. This would not affect the standard of treatment or current care you receive.

**What will happen to me if I take part?**

If you are interested to take part, then at first you will sign a consent form. Then, I will measure your height and weight to calculate your body mass index (BMI) to place you either obese (BMI ≥ 30) or non-obese group (BMI < 30). Finally, I will give you (whether you are obese or non-obese) a questionnaire about your current health status and current care and support you receive. Few questions are YES/ NO, but few questions need descriptive answers- no more than 1-2 lines. The whole questionnaires would roughly take 10-15 mins. I will write the answers for you. I will also inform your GP about your participation in this study. I will choose randomly any 10 participants for in depth interview to get more clearer idea about the health and social care practice.

**How long does the study last?**

The actual study would take place between December 2019 and December 2020. As mentioned above, your involvement should only last about 30-40 minutes.

**Where will my data go?**

All information which is collected about you will be kept strictly confidential in the password protected University server and only I will have access to it. Every questionnaire would denote individual as a case no. So, your name or hospital number won’t be written anywhere in the study file. All the computers storing patient data must meet special security arrangements.

**What are the potential disadvantages and risks of taking part?**

There are no significant risks associated with taking part. As your confidentiality will always be protected and I am going to denote you as a case no. in this research project.

**What are the possible benefits of taking part?**

As this study does not involve treatment, there are no direct benefits of taking part. The study, however, will provide useful insights into patients’ perspectives. It will also increase our understanding of the relationship between patients’ attitudes and expectation towards the health and social care received. Such information has the potential to benefit patient care in future.

**Will my taking part in the study be kept confidential?**

Yes, I will follow ethical and legal practice throughout this study. All information which is collected about you will be kept strictly confidential and only I and my supervisor will have access to it. Every questionnaire would denote individual as a case no. So, your name or hospital no. won’t be written anywhere in the study file.

**What are my choices about my patient data?**

You can stop being part of a research study at any time, without giving a reason, but the research team will keep the research data about you that they already have. I will manage your records in specific ways for the research to be reliable. This means that they won’t be able to let you see or change the data they hold about you. Research could go wrong if data is removed or changed.

**What happens to my research data after the study?**

The results of this study will form a major component of my final thesis. Depending on the findings, the study results will be presented at a conference or published. Your interview transcripts, after removing identifiable details, will be used for teaching, future research, and publication purposes. Researchers must make sure they write the reports about the study in a way that no-one can work out that you took part in the study.

Once they have finished the study, the research team will keep the research data for 5 years in the password protected University server, in case they need to check it. You can ask about who will keep it, whether it includes your name, and how long they will keep it.

Usually, your hospital or GP where you are taking part in the study will keep a copy of the research data along with your name. The organisation running the research will usually only keep a coded copy of your research data, without your name included. This is kept so the results can be checked.

If you agree to take part in a research study, you may get the choice to give your research data from this study for future research. Sometimes this future research may use research data that has had your name and NHS number removed. Or it may use research data that could show who you are. You will be told what options there are. You will get details if your research data will be joined up with other information about you or your health, such as from your GP or social services.

Once your details like your name were removed, other researchers won’t be able to contact you to ask you about future research.

Any information that could show who you are will be held safely with strict limits on who can access it.

You may also have the choice for the hospital or researchers to keep your contact details and some of your health information, so they can invite you to take part in future clinical trials or other studies. Your data will not be used to sell you anything. It will not be given to other organisations or companies except for research. No identifiable information of yours will be included in any of the reports. Having completed the research I will also share the executive summary of the study with the GP surgery, so that you get informed.

**Who has reviewed the study?**

All research in the NHS is looked at by independent group of people called a Research Ethics Committee, to protect your interests. This study was reviewed by the IRAS (Integrated Research Application System) for ethical approval and by my university research ethics committee.

**Will the use of my data meet GDPR rules?**

GDPR stands for the General Data Protection Regulation. In the UK we follow the GDPR rules and have a law called the Data Protection Act. All research using patient data must follow UK laws and rules.

Universities, NHS organisations and companies may use patient data to do research to make health and care better.

Universities and the NHS are funded from taxes, and they are expected to do research as part of their job. They still need to be able to prove that they need to use patient data for the research. In legal terms this means that they use patient data as part of ‘a task in the public interest’.

If they could do the research without using patient data, they would not be allowed to get your data.

Researchers must show that their research takes account of the views of patients and ordinary members of the public. They must also show how they protect the privacy of the people who take part. An NHS research ethics committee checks this before the research starts.

**What if I don't want my patient data used for research?**

You will have a choice about taking part in a non-clinical interview session. If you choose not to take part, that is fine.

In most cases you will also have a choice about your patient data being used for other types of research. There are two cases where this might not happen:

When the research is using anonymous information. Because it’s anonymous, the research team don’t know whose data it is and can’t ask you.

When it would not be possible for the research team to ask everyone. This would usually be because of the number of people who would have to be contacted. Sometimes it will be because the research could be biased if some people chose not to agree. In this case a special NHS group will check that the reasons are valid. You can opt-out of your data being used for this sort of research. You can ask your GP about opting out, or you can find out more at: https://www.hra.nhs.uk/information-about-patients/.

**Who can I contact if I have a complaint?**

If you want to complain about how researchers have handled your information, you should contact the research team. If you are not happy after that, you can contact the Data Protection Officer. The research team can give you details of the right Data Protection Officer.

If you are not happy with their response or believe they are processing your data in a way that is not right or lawful, you can complain to the Information Commissioner’s Office (ICO) (www.ico.org.uk or 0303 123 1113).

**Further information and contact details:**

For specific information about this project feel free to contact me – E-mail: 21374279@student.uwl.ac.uk.

Last Modified 4th December 2019
