# Supplementary material for: A qualitative study to examine hidden care burden for older adults with overweight and obesity in England
Source: PLoS One. 2025 Mar 19;20(3):e0320253. doi: 10.1371/journal.pone.0320253 (PMC11922259; doi:10.1371/journal.pone.0320253)
Supplement: S6 File — (DOCX) [file pone.0320253.s006.docx]

Semi-structured Questionnaires for Qualitative Interviews

(01/10/19, Version 0.2)

**Name of the GP surgery:** Addison House Surgery, Harlow

**Name of the interviewer:** Gargi Ghosh

**Date of Interview:**

**Participant serial number:**

| Age in years:  Gender: Male/ Female/ Other  Ethnicity:  Marital Status / Existing partner: Yes / No | BMI:  Height:  Weight: |
| --- | --- |

Questionnaire for participants (to be filled in either by yourself or by **Gargi Ghosh** as an interviewer):

1. Do you face any problem with any of the following activities in daily living? (Choose an option)

| - difficulty in dressing - difficulty in walking - difficulty in bathing/showering - difficulty in eating, such as cutting up foods - difficulty getting in and out of bed - difficulty using toilet including getting up or down - difficulty using map - difficulty recognising physical danger - difficulty preparing a hot meal - difficulty shopping for groceries - difficulty making phone calls - difficulty with communication - difficulty taking medications - difficulty working around house and garden - difficulty managing money, such as paying bills - None of the above - Other |
| --- |

If you choose other or a combination of more than one options from the above selection, please mention in the box below -

|  |
| --- |

If you face any problem with one or more of the above activities in daily living, how do you manage to do those activity/ activities? How do you feel about this?

|  |
| --- |

2. Do you use any of the following technologies to help you moving/daily living? (Choose an option)

| - A cane or walking stick - A Zimmer frame or walker - Wheelchair - Buggy/scooter - Special eating utensils - A personal alarm in event of fall - Elbow clutches - None of these above - Other |
| --- |

If you select a combination of more than one options from the above selection or other, please mention in the box below -

|  |
| --- |

If you use any of the above technologies or other to help you moving/daily living, please mention how do you feel about using that?

|  |
| --- |

3. What is your health in general? (Choose an option)

| Excellent | Very good | Good | Fair | Poor |
| --- | --- | --- | --- | --- |

If you choose fair or poor, please mention if any reason that you think of?

|  |
| --- |

| YES | NO |
| --- | --- |

4. Do you have any chronic illness? (Physical or mental impairment, or disability)? (Choose an option)

5. Which of the following score best describes how safe you feel at your own home? (Out of 10 score, where 0 is the worst and 10 is the best)

| 0 1 2 3 4 5 6 7 8 9 10 |
| --- |

Please mention if any reason that you think of behind the score you are choosing for?

|  |
| --- |

6. How satisfied are you with your life overall? (Choose an option)

| Fully satisfied | Partially satisfied | Unsatisfied |
| --- | --- | --- |

If you choose partially satisfied/unsatisfied, please mention if any reason that you think of-

|  |
| --- |

| YES | NO |
| --- | --- |

7. Are you currently receiving any social care support? (Choose an option)

If you select No, please mention if you think that you should receive one and why?

|  |
| --- |

(If Q7 marks -**Yes**, then please answer- (8a-8j)

8a. Who is the person/organisation providing you the care and support?

(Choose an option)

| - Husband/wife/partner - Children/grand children - Siblings - Other Relatives - Friend/neighbour - Homecare worker/home help/ personal assistant - Member of staff at care/nursing home - Member of reablement team - Warden/shelter housing manager/cleaner - Council handyman - other |
| --- |

If you choose other or a combination of more than one option from the above selection, please mention in the box below -

|  |
| --- |

8b. If you are receiving care from any family members/friends/Neighbor, how do you feel about having support from your family member?

(Choose an option)

| Fully satisfied | Partially satisfied | Unsatisfied |
| --- | --- | --- |

If you choose partially satisfied/unsatisfied, please mention if any reason that you think of-

|  |
| --- |

8c. If any of the following pay for your social care? (Choose an option)

| - Local authority/ social services or council pay - Spouse/partner - Other family member/friend - On your own |
| --- |

| YES | NO |
| --- | --- |

If you pay for your own care, is it stressful for you to pay for your own care? (Choose an option)

If your spouse/ partner/ other family member/ friend pays for your care, please mention how do feel about that?

|  |
| --- |

8d. Overall, how satisfied, or dissatisfied are you with the care and support services you receive? (Choose an option)

| Fully satisfied | Partially satisfied | Unsatisfied |
| --- | --- | --- |

If you choose partially satisfied/unsatisfied, please mention if any reason that you think of-

|  |
| --- |

8e. Whether help received meets your needs? (Choose an option)

| - Hardly ever meets the needs - Sometimes meets the needs - Usually meets needs - Meets needs all the time |
| --- |

| YES | NO |
| --- | --- |

8f. Are you happy with the amount of time your carer/family member or friend spend with you for the care purpose? (Choose an option)

8g. Are you satisfied the way the care and support services help you keeping clean and presentable in appearance? (Choose an option)

| Fully satisfied | Partially satisfied | Unsatisfied |
| --- | --- | --- |

If you choose partially satisfied/unsatisfied, please mention if any reason you think of-

|  |
| --- |

8h. Are you satisfied the way the care and support services help you with your food and drink? (Choose an option)

| Fully satisfied | Partially satisfied | Unsatisfied |
| --- | --- | --- |

If you choose partially satisfied/unsatisfied, please mention if any reason you think of-

|  |
| --- |

8i. Are you satisfied the way the care and support services help you in feeling safe? (Choose an option)

| Fully satisfied | Partially satisfied | Unsatisfied |
| --- | --- | --- |

If you choose partially satisfied/unsatisfied, please mention if any reason you think of-

|  |
| --- |

8j. Which of these statements best describes how the way you are helped and treated makes you think and feel about yourself? (Choose an option)

| Excellent | Very good | Good | Fair | Poor |
| --- | --- | --- | --- | --- |

Any comment, if you choose fair or poor:

|  |
| --- |

9. What do you want more for your own social care?

|  |
| --- |

10. If there is anything else you would like to tell us, please write in the space below. We shall be very interested to read what you have to say.

|  |
| --- |

| **Perceived Outcomes:** |
| --- |
| - To explore the demand of healthcare usage of obese older adults: Q1 |
| - To inquire the relationship between high Body mass index and social care needs in older adults: Q3 to Q7 - To explore the need of technologies: Q2 |
| - To explore the need by health status: Q1, Q3, Q4 |
| - To explore the need of feeling safe: Q5. |
| - To explore the need of life satisfaction/quality of life/wellbeing: Q6 |
| - To examine the unmet needs for social care of obese older adults: Q8 to Q10 |
