# Supplementary material for: A qualitative study to examine hidden care burden for older adults with overweight and obesity in England
Source: PLoS One. 2025 Mar 19;20(3):e0320253. doi: 10.1371/journal.pone.0320253 (PMC11922259; doi:10.1371/journal.pone.0320253)
Supplement: Attachment — Submitted filename: PONE-D-24-21647_reviewer.pdf [file pone.0320253.s007.pdf]

# PLOS ONE

## Factors Influencing the Hidden Burden of Care for Older Adults, with Overweight and Obesity, in England: A Qualitative Study

--Manuscript Draft--

| | |
|---|---|
| **Manuscript Number:** | PONE-D-24-21647 |
| **Article Type:** | Research Article |
| **Full Title:** | Factors Influencing the Hidden Burden of Care for Older Adults, with Overweight and Obesity, in England: A Qualitative Study |
| **Short Title:** | The Unmet care need for Older Adults, with Overweight and Obesity |
| **Corresponding Author:** | Gargi Ghosh, PhD<br>Anglia Ruskin University<br>Chelmsford, Essex UNITED KINGDOM |
| **Keywords:** | older people, elderly, obesity, social care, unmet needs, England |
| **Abstract:** | Objectives<br>This qualitative study aimed to examine the association between overweight or obese older adults living in England, and their formal and informal social care needs.<br>Methods<br>Participants were recruited from a local NHS health centre in London England using a purposive sampling strategy to the point of analytical saturation. A total of 45 participants were invited and of these 33 participants were eligible to take part. A semi-structured interview was conducted, and a qualitative thematic analysis was undertaken.<br>Results<br>The study found that older adults, who are overweight or obese, were more likely to have physical health problems and problems with mobility. They were more likely to have informal voluntary care and support rather than formal social care support. They also had a weaker social support network, were more isolated and frustrated, lacked housing adaptations, felt unsafe, felt they were a burden to their families and felt discriminated against by the wider community. Care and support need If not met, then these are likely to generate or widen health inequalities over time.<br>Conclusions<br>Older adults in England, who are overweight and obese, experience a range of physical, particularly mental health and wellbeing issues. This represents a currently hidden, growing population group that will likely require formal social care support in the coming years. National and local policy and decision-makers will need to address this unmet care need to reduce the potential burden of health care costs and poor physical and mental health and wellbeing. |
| **Order of Authors:** | Gargi Ghosh, PhD |
| | Hafiz TA Khan, PhD |
| | Salim Vohra, PhD |
| **Additional Information:** | |
| **Question** | **Response** |
| **Financial Disclosure**<br><br>Enter a financial disclosure statement that describes the sources of funding for the work included in this submission. Review the submission guidelines for detailed requirements. View published research articles from *PLOS ONE* for specific examples. | The author(s) received no specific funding for this work. |

*Powered by Editorial Manager® and ProduXion Manager® from Aries Systems Corporation*

| | |
|---|---|
| This statement is required for submission and **will appear in the published article** if the submission is accepted. Please make sure it is accurate.<br><br>**Funded studies**<br>Enter a statement with the following details:<br>• Initials of the authors who received each award<br>• Grant numbers awarded to each author<br>• The full name of each funder<br>• URL of each funder website<br>• Did the sponsors or funders play any role in the study design, data collection and analysis, decision to publish, or preparation of the manuscript?<br><br>Did you receive funding for this work? | |
| **Competing Interests**<br><br>Use the instructions below to enter a competing interest statement for this submission. On behalf of all authors, disclose any competing interests that could be perceived to bias this work—acknowledging all financial support and any other relevant financial or non-financial competing interests.<br><br>This statement is required for submission and **will appear in the published article** if the submission is accepted. Please make sure it is accurate and that any funding sources listed in your Funding Information later in the submission form are also declared in your Financial Disclosure statement.<br><br>View published research articles from *PLOS ONE* for specific examples. | The authors have declared that no competing interests exist. |

Powered by Editorial Manager® and ProduXion Manager® from Aries Systems Corporation

| | |
|---|---|
| **NO authors have competing interests**<br><br>Enter: *The authors have declared that no competing interests exist.*<br><br>**Authors with competing interests**<br><br>Enter competing interest details beginning with this statement:<br><br>*I have read the journal's policy and the authors of this manuscript have the following competing interests: [insert competing interests here]*<br><br>* typeset | |
| **Ethics Statement**<br><br>Enter an ethics statement for this submission. This statement is required if the study involved:<br><br>• Human participants<br>• Human specimens or tissue<br>• Vertebrate animals or cephalopods<br>• Vertebrate embryos or tissues<br>• Field research<br><br>Write "N/A" if the submission does not require an ethics statement.<br><br>General guidance is provided below. Consult the submission guidelines for detailed instructions. **Make sure that all information entered here is included in the Methods section of the manuscript.** | 1. HRA and Health and Care<br>Research Wales (HCRW)- "I am pleased to confirm that HRA and Health and Care Research Wales (HCRW) Approval was given for the above referenced study, on the basis described in the application form, protocol, supporting documentation and any clarifications received".<br>IRAS project ID: 253586<br>Protocol number: 21374279<br>REC reference: 19/LO/1093<br>Sponsor: University Of West London<br><br>Written signed consents were obtained. |

| | |
|---|---|
| **Format for specific study types**<br><br>**Human Subject Research (involving human participants and/or tissue)**<br>• Give the name of the institutional review board or ethics committee that approved the study<br>• Include the approval number and/or a statement indicating approval of this research<br>• Indicate the form of consent obtained (written/oral) or the reason that consent was not obtained (e.g. the data were analyzed anonymously)<br><br>**Animal Research (involving vertebrate animals, embryos or tissues)**<br>• Provide the name of the Institutional Animal Care and Use Committee (IACUC) or other relevant ethics board that reviewed the study protocol, and indicate whether they approved this research or granted a formal waiver of ethical approval<br>• Include an approval number if one was obtained<br>• If the study involved *non-human primates*, add *additional details* about animal welfare and steps taken to ameliorate suffering<br>• If anesthesia, euthanasia, or any kind of animal sacrifice is part of the study, include briefly which substances and/or methods were applied<br><br>**Field Research**<br><br>Include the following details if this study involves the collection of plant, animal, or other materials from a natural setting:<br>• Field permit number<br>• Name of the institution or relevant body that granted permission | |
| **Data Availability**<br><br>Authors are required to make all data underlying the findings described fully available, without restriction, and from the time of publication. PLOS allows rare exceptions to address legal and ethical concerns. See the PLOS Data Policy and FAQ for detailed information. | Yes - all data are fully available without restriction |

| | |
|---|---|
| A Data Availability Statement describing where the data can be found is required at submission. Your answers to this question constitute the Data Availability Statement and **will be published in the article**, if accepted.<br><br>**Important:** Stating 'data available on request from the author' is not sufficient. If your data are only available upon request, select 'No' for the first question and explain your exceptional situation in the text box.<br><br>Do the authors confirm that all data underlying the findings described in their manuscript are fully available without restriction? | |
| **Describe where the data may be found in full sentences. If you are copying our sample text, replace any instances of XXX with the appropriate details.**<br><br>• If the data are **held or will be held in a public repository**, include URLs, accession numbers or DOIs. If this information will only be available after acceptance, indicate this by ticking the box below. For example: *All XXX files are available from the XXX database (accession number(s) XXX, XXX.).*<br>• If the data are all contained **within the manuscript and/or Supporting Information files**, enter the following: *All relevant data are within the manuscript and its Supporting Information files.*<br>• If neither of these applies but you are able to provide **details of access elsewhere**, with or without limitations, please do so. For example:<br><br>*Data cannot be shared publicly because of [XXX]. Data are available from the XXX Institutional Data Access / Ethics Committee (contact via XXX) for researchers who meet the criteria for access to confidential data.*<br><br>*The data underlying the results presented in the study are available from (include the name of the third party* | All relevant data are within the manuscript and its Supporting Information files. |

| | |
|---|---|
| *and contact information or URL).*<br>• This text is appropriate if the data are owned by a third party and authors do not have permission to share the data.<br><br>* typeset | |
| Additional data availability information: | |

Manuscript

Click here to access/download;Manuscript;Manuscript Final.docx

**Title:** Factors Influencing the Hidden Burden of Care for Older Adults, with Overweight and Obesity, in England: A Qualitative Study

**Authors**

1. [1]Dr Gargi Ghosh,

The University of West London, Boston Manor Road, Brentford TW8 9GB, UK

ORCID id- 0000-0003-0876-5094

E: gargi.ghosh@aru.ac.uk,

2. Professor Hafiz T.A. Khan,

The University of West London, Boston Manor Road, Brentford TW8 9GB, UK.

E: hafiz.khan@uwl.ac.uk

3. Dr Salim Vohra,

The University of West London, College of Nursing, Midwifery and Healthcare, Paragon House, Boston Manor Road, Brentford, Middlesex TW8 9GA, UK.

E: salim.vohra@uwl.ac.uk

**Statements and Declarations**

This research did not receive any specific grant from public, commercial, or not-for-profit funding agencies.

**Declarations of interest: none.**

[1] Anglia Ruskin University, Faculty of Health, Education, Medicine & Social Care (HEMS). Chelmsford, CM1 1SQ, UK

## Acknowledgement

This research is an outcome of the first author's PhD study (Ghosh et al., 2021) at the University of West London, UK.

## ABSTRACT

**Objectives**

This qualitative study aimed to examine the association between overweight and older adults with obesity living in England, and their formal and informal social care needs.

**Methods**

Participants were recruited from a local NHS health centre in London England using a purposive sampling strategy to the point of analytical saturation. A total of 45 participants were invited and of these 33 participants were eligible to take part. A semi-structured interview was conducted, and a qualitative thematic analysis was undertaken.

**Results**

The study found that older adults, who are overweight or obese, were more likely to have physical health problems and problems with mobility. They were more likely to have informal voluntary care and support rather than formal social care support. They also had a weaker social support network, were more isolated and frustrated, lacked housing adaptations, felt unsafe, felt they were a burden to their families and felt discriminated against by the wider community. Care and support needs If not met, then these are likely to generate or widen health inequalities over time.

**Conclusions**

Older adults in England, who are overweight and obese, experience a range of physical, particularly mental health and wellbeing issues. This represents a currently hidden, growing population group that will likely require formal social care support in the coming years. National and local policy and

decision-makers will need to address this unmet care need to reduce the potential burden of health care costs and poor physical and mental health and wellbeing.

Keywords: older adults, elderly, obesity, social care, unmet needs, England

## INTRODUCTION

As numbers of older adults (defined as those aged 65 years and over) increase as populations age globally, the disease burden of those with chronic conditions, i.e., multi-morbidity, has become a significant concern internationally and in England (Han et al., 2011; Local Government Association, 2020). A report by the Office for National Statistics (ONS, 2013) stated that in Great Britain (England, Scotland, and Wales), 36% and 20% of adults reported having a disability or other limiting long-term condition, respectively. Globally, obesity is the cause of more than 3.4 million deaths, 4% of life years lost and at least 4% of 'Disability-Adjusted Life Years' (Ng et al., 2014). The prevalence of obesity among adults in the most disadvantaged areas is approximately double that in the least deprived area, 36% compared to 20% (Batterham, 2020).

Yet an often overlooked chronic condition in older people is being overweight or obese, which can lead to functional impairments (Tomlinson et al., 2016; Alexandre et al., 2018). A cross-sectional study by Sørbye et al. (2007) on care needs for older women aged 65 years and above with morbid obesity evaluated that extremely obese women require more help with their personal care than their normal-weight counterparts. Obesity is also often associated with social discrimination (related to age and obesity), stigma and body image distortions in older adults (Trull et al., 2012; Abdelaal et al., 2017). A report by Wharton et al. (2020) stated that the association between obesity with stigma and social bias is one of the essential contributors to increased morbidity and mortality in older adults regardless of the weight categories. Social discrimination and stigma can increase the risk of low self-esteem, loneliness, depression, problems with interpersonal relationships, communication, mood changes and poor health behaviours among older adults (Djalalinia et al., 2015; Age UK, 2019).

An individual’s health and wellbeing and physical bodily mobility directly defines their social care needs, and the support required to help them live, as independently as possible, in their own homes. These needs are complex and multi-dimensional and include the functional activities that we all carry out in normal daily life, for example, washing and dressing. At least five different types of social care needs can be identified: normative, felt, expressed, comparative and technical (Bradshaw, 2013; Kettner et al., 1990). Normative needs are defined and set by professionals according to, nationally determined, desired standard of care. Felt needs refers to what people want, the expectation of a social care service user based on their own beliefs about their needs. An expressed need refers to a demand for social care expressed by an individual to a service, which can be unmet, partially met or fully met. Comparative need compares the difference in the range and intensity of service received by two service users. Technical need occurs when a new technical breakthrough leads to a new type of social care service or a more effective version of an existing service (Vlachantoni et al., 2011). Of these, in relation to the social care needs of older people who are overweight or obese, the two key ones are felt needs and expressed needs that are unmet, partially met or fully met.

In England, social care for older adults is provided through three community-based support systems: formal state support, formal private support and informal family support. Formal state support comprises the public social care services provided for free or free up to some level. Formal private support is additional social or nursing care that an individual, or their family, pays for from their own financial resources, in addition to or instead of that provided by formal state support. Informal family support is continuous support, often 24/7, provided by family members. Often older people have support from all three systems.

The difference between an individual's need for help with daily living and the amount of social care they actually receive is their unmet social care need (Copley et al., 2017). Using Health Survey for England (HSE) 2013 data, Thompson et al. (2014) found that a greater social care need was reported among older adults than the help and care actually received. A study by Brimblecombe et al. (2016) on carers and care recipients reported that about 47% of care recipients expressed that they required more services to satisfy their needs. Other recent studies have also found evidence of unmet care need among older adults in England (e.g.. Age UK, 2019; Dunatchik et al., 2019). Often such need relates to functional activities: NHS England (NHSE, NHS Digital, 2020) reported that there were 19% of men and 28% of women over 65 years had an unmet need with at least one activity of daily living (ADL) difficulty, and 12% of men and 15% of women had an unmet need with at least one instrumental ADL (IADL) difficulty.

Over the last two decades, cuts to adult social care budgets in England have reduced the capacity of social care services to meet the current and future needs of older people as more and more restrictions have been placed on who gets formal state support (Vlachantoni et al., 2011). To receive formal state support, an individual must meet certain eligibility criteria. These include an individual's marital status, living arrangements, physical and mental health status, ability to use technologies, and finally their socio-economic status (Vlachantoni et al., 2015). These criteria relate to whether an individual has access to informal social care (a spouse or children living with them), the ability to pay for formal state or formal private support, and the physical functioning to enable them to use mechanical and electronic aids. Another factor in unmet social care need is either an inability of health and social care services to reach the target population, or that individuals in need somehow drop out of the formal state health and social care system (NHS England, 2017).

Given the significant and growing gaps in formal state social care support there is increasing dependence on the informal family and formal private social care systems. This is particularly so when some or all of an individual's need remains outside the entitlement criteria of formal state social care support (Vlachantoni et al., 2011). The majority of social care needs of older people are met through informal family support. Given this, it is unsurprising that older people are net providers of social care support (Haberkern et al., 2012, p.194). However, this reliance on informal care is leading to an increase in the burden of disease on informal family carers and an increase in hospital admissions as older people with long-term conditions and unmet social care needs are not able to manage their conditions.

The Commission for Social Care Inspection (2008) considers ‘unmet need’ a vital and challenging part of social care policy, and the measurement of unmet care need is key in evaluating the efficacy of social care provision. The Care Act (Department of Health (DOH), 2014) in England demands greater focus on the prevention, reduction and delay of social care needs for all people. It outlines three stages of prevention.. Primary prevention seeks to prevent or delay the development of social care needs in individuals without any pre-existing social care needs. Secondary Tertiary prevention focus on reducing existing social care needs and delaying progression to more intensive social care needs. This is achieved by providing adaptations and aids, for example, handholds, mobility aids, and disabled shower rooms, to maintain independence in the activities of daily living (Dunatchik et al., 2019).. Identifying existing needs (even less important or low-level needs) at the developmental level is beneficial to minimise demand on future social care services and maximise its effectiveness.

The objective of the present study was to examine the association between older adults, living in England, who are overweight or obese, and their formal and informal social care needs.

Exploring unmet social care needs for older adults with long-term health conditions, and estimating the hidden burden of disease and healthcare costs is important to enhance their quality of life, to minimise demand for future formal and informal social care support, and to maximise the effectiveness of formal state, private and informal family social care support.

## METHODS

The study is designed to have purposeful sampling. Therefore, a semi-structured one-to-one interview, or "guided conversation," was conducted with potential participants who met the study's inclusion and exclusion criteria. Each eligible participant interested in the study received an information sheet and consent form prior to the interview. Written and signed consent was obtained from all participants before participation. The recruitment period and primary data collection for this study began on July 3, 2020, and concluded on December 1, 2020.

### Underpinning Theory

The key theories that underlie the findings of the present study are firstly, seeing obesity through a life course approach and social cognitive theory lens. Secondly, understanding disability through a human rights approach and social model of disability. Thirdly, framing wellbeing is about helping individuals to recognise the abilities they have, not their disabilities, and feeling empowered and enabled. Lastly, appreciating that how individuals and organisations perceive need is multi-layered and includes felt need, expressed need, and unrecognised need.

Examining individuals' lives within the context of social change, and structural and cultural circumstances is called the life course approach (Elder et al., 2003). The life course approach "explains ways in which different factors may act to cause chronic diseases across the life course: they focus on a critical period (with or without effect modifiers), an accumulation of risk model and a chain of risk

model" (Jacob et al., 2017, p. 7). It was developed from evidence that early life experiences had profound health effects in later life (the Barker Hypothesis) (Edwards, 2017). Therefore, a life course approach needs to be taken to prevent disease and protect, promote, and improve health and wellbeing (Rashbrook, 2019). Elderly or ageing is, therefore, one pivotal phase of developmental change in the life course particularly in countries like the UK where the older adult population is increasing over time (Age UK, 2014). They are also more vulnerable to chronic diseases due to the biological ageing process and the potential for an increase in unhealthy behaviours in old age. Research by Age UK (2019) found that for older adults, the effects of inadequate social connection are as dangerous as the effects of inactivity, obesity, and smoking. So, there is an association between a lack of social connection and an increased risk of chronic disease in older adults (Age UK, 2019). In the context of the present study, overweight and obesity, health and wellbeing, and social care needs in older adults must take account of the life course of an older adult and how they may have experienced a cumulative burden of adverse social, economic, physical environment and social impacts during childhood, adolescence, and adulthood.

Social cognitive theory (SCT) states that "learning occurs in a social context with a dynamic and reciprocal interaction of the person, environment, and behaviour" (LaMorte, 2019, para. 5). An individual's health behaviour, therefore, depends on the impact of an individual's past and present experiences, environmental factors, and the act of others (Bandura, 2004). In the context of the present study, the last point about the actions of others (informal, formal public or state, and paid private social care) is an important issue to bear in mind and how an individual's life course (past and present experiences) alongside their current social and physical environment (health inequalities) is likely to influence their level of obesity, their wellbeing, and their level of social care need.

In the context of the present study, whether, and how, social care empowers and enables individuals to take charge of their own physical, mental, and social health and wellbeing, i.e., so that they stay independent for as long as possible, is an important issue to be considered.

In contrast, from a medical model of disability perspective, if obesity is considered a disease or something is ‘wrong’ with that individual, then an individual with disabilities feels “excluded, undervalued, pressured to fit a questionable norm, and/or treated as if they were globally incapacitated” (Goering, 2015, p.134). The social model, therefore, assists us in rethinking the term- disability and acknowledging the roadblocks that force an individual with functional difficulties or ill health to be impaired and dependent. However, as discussed before, helping a person to overcome societal impairments have an association with significant cost implications and workforce. Hence it demands an improvised public health policy and planning. The human rights model of disability aims at the fundamental human rights of a person with disabilities (Degener, 2014), which is primarily grounded on the social model of disability. According to the model, disability is viewed as a shift from an individual's everyday health and illness status. Fundamentally, the purpose of the model is to focus on preserving an individual's self-worth and dignity. In addition, this model considers an individual's medical needs and the nature of the illness (Degener, 2014). In contrast to the social model, “the human rights model encompasses the values for a disability policy that acknowledges the human dignity of disabled persons” (Degener, 2014, p.6). A report by LGA (2020) informed that the Equality Act of 2010, is yet to be safeguarded by a person's weight category. Anyhow, perhaps there is a legal provision against unfairness for an individual with obesity, given that an increasing degree of body weight adversely affects an individual's ability to continue daily activities (LGA, 2020). Even so, an appeal produced by the United Kingdom Employment Appeal Tribunal database (2013) emphasised considering 'obesity discrimination' as 'disability discrimination’.

The critical disability theoretical model raises questions on the dualism between impairment and disability, and it is grounded on the social model and human rights model of disability. A paper presented by Hosking (2008, p.5) explained that “Critical disability theory centres

disability as it compares liberalism's norms and values with their actualisation in the daily life of disabled people". Primarily, the model is based on the principles that assist any country's law and legal system to view and respond towards a disabled person. In addition, most of the disability models are partially based on the 'structural and political economy' theory of health inequalities. Mccartney (2017, p.19) reported that "Those with most resources are always the healthiest, regardless of their behaviours".

In the context of the present study, disability is theoretically framed using a human rights approach and the social model of disability.

Furthermore, it is important to discuss what 'need' is. Davis (1955) defined the need as a state of individuals' mind that encourages them to choose amidst various available therapeutic resources. The term 'need' is, therefore, a complex concept interpreted and defined in many ways that can change over time. Thus, each of the definitions of need has been developed to improve quality-of-service delivery to meet individuals' expectations and demands according to their physical, mental, and social health and wellbeing.

In the context of social care needs, it is vital to mention Bradshaw's taxonomy (Bradshaw, 2013), which compares four different aspects of social needs an individual can have: normative, felt, expressed and comparative needs. While professionals can set normative needs according to the desired standard of care, a felt need refers to 'what people want, or the expectation of a service user based on their own beliefs of need. An expressed need refers to a demand, which is unmet, partially met and fully met. Finally, the comparative need is defined as comparing the degree of service received by two service users. Along with Bradshaw's taxonomy of social care provision, there is a shred of evidence (Kettner et al., 1990) of another important social care need: technical need. A Centre for Population Change (CPC) report by Vlachantoni (2011, p.4) informs "technical need occurs when existing provision is made more effective, or a new

kind of provision is invented, in which case a need for a new kind of solution arises". In the context of the present study, it is important to recognise that need is complex and multi-dimensional, it includes felt need and expressed need that is unmet, partially met or fully met.

**Measurement of variables**

**Obesity**

To determine unmet care needs, weight was categorised into five groups: normal weight, overweight, moderate or class I obesity, severe or class II obesity, and morbid or class III obesity. This was based on the three categories of obesity developed by WHO: obesity class I (BMI = 30-34.9 kg/m2), obesity class II (BMI = 35-39.9 kg/m2) and obesity class III (BMI ≥ 40 kg/m2) (WHO, 2020).

**Unmet care needs for social care and support**

The present study defines unmet care needs as any needs that an individual identifies, related or unrelated to functional or mobility impairment, that are not cared for, or supported enough by, existing social care services. This definition is broader than other definitions and encompasses the activity-based aspects of need (the ADL & IADL) along with other key aspects of life such as a sense of purpose, safety, loneliness, and social contact, so that as many hidden unmet needs for social care can be identified.

Vlachantoni et al. (2011) argued that unmet care need (U) is the gap between demand and supply. Where demand is either impairment in mobility or inability to perform one of the tasks under ADLs or IADLs activities (i.e., service-based or normative needs) and supply is the informal family, formal public or state, and formal paid private care. Dunatchik et al. (2019) however argue that applying a task-focused approach (ADL and IADL activities based) to evaluate unmet care needs "… may underplay the importance of other aspects of life such as social contact and maintaining a sense of purpose" (page 196). In addition, exploring 'low-

level needs' which may not be a function or impairment-related care needs is an issue highlighted in the Care Act Guidance Secondary Prevention Plan (Department of Health and Social Care, 2021). According to the Care Act (DOH, 2014), it is crucial to prevent care needs by preventing the development of low-level needs.

**Unmet care needs for social care and support: A conceptual model**

An unmet care need for social care and support was conceptualised (See Figure 1) to fulfil the present study's objectives. The purpose of the model is to predict a range of needs that are required for formal social care. This model helps to identify gaps in service provision, even among those who do not meet the eligibility criteria (by Local Authorities of England) for current social care support and those who are eligible but may not realise their needs. However, the present study might have overestimated the amount of unmet care needs. Identifying the potential needs at the developmental level and exploring existing unmet care needs due to the lack of service provision may help to plan ahead and protect society from higher health and social care costs in the long term. This study also uses a qualitative research methodology on a small sample to evaluate the older adult's perspectives and generate new knowledge about the types and levels of unmet needs older adults have, rather than estimate the number of people having unmet needs of various types.

In this model (See Figure 1), the Y-axis denotes social care support (S), and the X-axis refers to social care needs (N). According to the model, from the centre (0,0), social care support increases along the Y-axis, and social care needs increase along the X-axis. Therefore, according to the need and support received, the population are divided into five groups. Group A represents older adults who do not have any need (in terms of functional and mobility impairments and any other individual felt needs), but they receive support (whether informal family, formal state or formal private paid-for care or a combination). Group B represents the older adults who do not have any needs and are not receiving any social care. Group C1

represents older adults who have at least one need (either social care service-defined or individual-felt), and their needs are fulfilled by the social care they receive. Group C2 represents older adults who are not satisfied with the support they are receiving according to their felt needs. Hence, they have existing unmet felt needs and unmet needs for social care. Group D represents older adults who have at least one need but are not receiving any social care. Therefore, this group of people has unmet felt needs and unmet needs for social care. The present study's focus is to collect primary data about two groups of older adults in the mode, Groups C2 and D, and explore the association between unmet felt needs and unmet needs for social care by the degree of BMI in older adults.

**Figure 1: Conceptualised model on unmet care needs for social care and support**

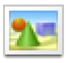

Figure 1.png

Constructed by Ghosh (2022)

**Ethical Consideration**

Ethical approval was obtained jointly from the University of West London ethical approval board (College of Nursing, Midwifery and Healthcare (CNMH) Research Ethics Panel) and the UK Health Research Authority (Health Research Authority, HRA, 2018). In the UK, where the present study is undertaken, all health, community, and social care research, involving human participants, must apply for ethical approval through the HRA's Integrated Research Application System (IRAS). A research application form was submitted to the HRA through a single system IRAS for both HRA approval and for Research Ethics Committee (REC) review. Therefore, the risk of adverse consequences of exploring sensitive topics was avoided or minimised among this potentially socially disadvantaged population group.

### The venue

Convenience sampling was used to identify a single NHS GP surgery as the venue for the present study. Choosing a GP surgery provided a private, quiet, non-threatening environment that encouraged participants to engage in a rich and in-depth conversation, besides, the surgery provided easy access for participants. The surgery also had essential amenities such as a water dispenser and toilets.

### Privacy and confidentiality

The participants' General Practitioners (GPs) were informed of their involvement in this study to ensure that GPs could discuss and follow up with the participant after the interview. No identifiable information was taken from the participants (not even their names/hospital numbers/date of birth). All audio recordings were saved in a password-protected computer as digital files and destroyed once the audio transcriptions had been completed and written up. Direct quotations from the respondents will be used for publication/ conference presentation, but any person's name/company name/organising committee name was removed from the written transcriptions. Participants were assigned a unique numerical case number to ensure anonymity. Only the student researcher (then) and an independent researcher from the UWL heard the recordings and read the transcripts to verify the transcription. The field notes and the student researcher's reflective diary were shared with doctoral supervisors.

### Participant welfare

During the design of the study, no major adverse risks were identified for the participants taking part in a one-to-one semi-structured interview. However, it was considered that some questions or topics might be more emotional than others, for example, talking about the coping strategies and the support or care they had or had not received related to their increasing weight or

complex morbidity. Therefore, it was decided, and agreed by the ethics committees, that if any participants find any discussions upsetting, then the interview would be ended and the interviewer would give the participant time to calm down, discuss their mood with themselves or call someone else and offer them the opportunity to withdraw from the interview. It was anticipated that the length of the interview could be a potential concern for participants. Therefore, before beginning the consenting procedure, the likely length of the interview (30 minutes) was mentioned both verbally and in writing as part of the participants' information sheet. Finally, due to the Coronavirus Disease (COVID-19) pandemic, it was essential to undertake all the necessary measures to ensure participants' COVID safety. Hence, before bringing the participants into the interview room and after they left the interview room, the following measures were undertaken, such as wearing musk and plastic apron, sanitising hands/chair /pen/ door handles before and after each interview, maintaining a 2-meter distance, and keeping the windows open during the interview. In addition, at the beginning of every day the temperature of each recruiting GP surgery staff member, the student researcher and each participant were undertaken, and questions about symptoms of COVID were asked. If the student researcher, participants, or GP staff member had been diagnosed positive for COVID, then the interview would be cancelled, and the participants would be allowed to go home if they felt okay, or a family member or carer would be asked to escort the participant home.

**Researcher's safety**

A risk assessment was carried out to explore any potential risk to the student researcher in carrying out the one-to-one semi-structured interview in a closed room of the recruiting NHS GP surgery. All GP surgery staff and the local research lead were informed about the location of the interview room at the beginning of every planned interview day. The student researcher also kept their fully charged mobile phone in the interview room. In addition, while being aware of the NHS lone worker's policy as the research was conducted inside a GP surgery it was not

judged necessary to have a chaperone. Lastly, due to the COVID pandemic, the safety measures discussed in the previous section were also followed by and applied to, the student researcher.

**Debriefing and feedback**

After completing a one-to-one semi-structured interview, the recording device was turned off, and each participant was encouraged to ask further questions which were then answered by the student researcher. In addition, the participants were reminded about the ways the study findings would be disseminated (as discussed in a previous section). Finally, all the participants were provided with the student researcher's university student email address, and they were informed that they were always welcome to discuss any future concerns that they might have regarding their participation in this study.

**Data collection methods**

Data was collected through one-to-one guided interviews with the help of an interview guide. The interview duration ranged from 30-40 mins. A notice stating 'interview in progress' was hung from the interview room door handle. Prospective participants were asked for written consent followed by a full verbal explanation of the study, and the opportunity was given to read the participants' information sheet. Two participants requested to be withdrawn from the study before signing their consent forms. One participant stated, "I am in a rush", as she had another appointment to attend. Another participant considered the length of the interview to be too long. All the participants were offered either an in-person interview on a scheduled day or a phone interview to be conducted later. Family members and the carers were asked to wait outside in the waiting area lounge. Out of the total 33 cohorts, 5 participants requested a phone interview. All phone interviews were carried out using the surgery's phone. All participants were asked for their current disability and health status and demographic details. All interviews

were digitally recorded with permission being asked for from participants as part of the opening interview session. In addition, field notes were taken with pen and paper for each participant.

A reflective diary was maintained to record the experiences of the student researcher and memos were produced after interviewing each participant. Last but not the least, all participants were debriefed after the completion of the interviews.

**Informed consent**

Each suitable participant interested in taking part in this study was given a participants' information sheet and consent form before starting the one-to-one semi-structured interview. Every participant had to give signed consent before participating in the study. The study purpose, design, and involvement were discussed with the interviewer, that is the student researcher. All participants were reassured that their participation was voluntary, that the care they would typically receive would not be affected by their decision to participate or not in the research and that they had the right to withdraw their consent at any time both for the interview and any data already collected. In addition, the participants were informed that their personal identity would be protected; all data entered in this study would be by case numbers, not names. Finally, the participants were informed that an executive summary of the study findings would be shared with the participating NHS GP surgery (recruitment site) either by email/ letter or poster following the completion of the study and that they could receive a copy if they so wished directly. The participants were also asked and consented to their interview being used for teaching, future research, and publication after removing their identifiable details. Each participant was given a copy of the signed consent form, and one copy of the consent form was given to the local collaborator or local research lead to be placed in the participants' file.

**Semi-structured interviews**

Adopting semi-structured one-to-one interviews as a data collection is ideal “to explore participant thoughts, feelings and beliefs about a particular topic and to delve deeply into personal and sometimes sensitive issues” (DeJonckheere and Vaughn, 2019, p. 1). Therefore, this data collection method was well-suited to collect relevant, distinctive, and meaningful stories of participants' life experiences and aspirations and concerns.

For the current study, an interview guide was prepared to guide the interview to make effective use of time. According to the study context and objective, a validated questionnaire from ELSA (freely available) was used to form a loosely structured interview guide. Each question was supplemented by open-ended follow-up and probing questions that were dependent on the interviewee's response. Specific questions focused on weight-related perceived barriers in terms of disability, health and wellbeing and social care support and their perception of their coping strategies. The term ‘care and support’ was used throughout the interviews to assess the gaps between demand and service received.

The interview guide was reviewed by several reviewers as part of the ethics committee's approval, along with the student researcher’s supervisors. The interview guide is available on request from the student researcher.

**Sampling strategy**

Due to the COVID-19 pandemic, the participants had limited access to GP surgery, which meant a change in the original representative sampling strategy to a purposive sampling strategy to collect this qualitative data. Initially, the study was designed to have two closely overlapping stages. The first stage would be a semi-structured questionnaire given to randomly selected potential participants divided into two groups, obese and non-obese, for face-to-face and one-to-one real-time conversation and an in-depth one-to-one interview would follow with a smaller sample of participants randomly drawn from these two groups. However, given the

COVID-19 pandemic, a purposeful sampling strategy minimised contact between the interviewer, participants and GP surgery staff. As the qualitative inquiry is used to evaluate and measure the older adults' perceptions of whether providing social care support in any form is effective or not was judged to be acceptable. According to the study objective, the purpose was to find the most significant degree of information till no new information was generated (data saturation). Therefore, sampling was done to the point of redundancy. According to Patton (2002, p. 244), "There are no rules for sample size in qualitative inquiry".

**Eligibility criteria**

The following eligibility criteria were used for the recruitment process:

**Inclusion criteria:**

The population group to be included in the study satisfied the following criteria-

- Older adults of 50 years and over
- Older adults BMI ≥ 18.5 kg/m2
- Can speak and understand English.
- Older adults visiting minor illness clinics.
- Having the ability to deliver informed consent according to the UK Mental Capacity Act (Gov.UK, 2005)

**Exclusion criteria:**

Patients with a history of any of the following were excluded-

- Cannot speak and understand English.
- Advanced-stage dementia

- Individuals with severe or profound ID (intellectual disabilities)
- Individuals with Prader Willi syndrome, Cohen syndrome or Bardet-Biedl syndrome.

**Recruitment of participants**

Prospective 33 participants for the semi-structured interviews were recruited by me as the student researcher. Individuals who visited the surgery for flu vaccination and satisfied the inclusion and exclusion criteria set for this study were considered potential participants. Every Tuesday, except a few Tuesdays, either the surgery was closed due to the current pandemic, or the local collaborator was on leave, older adults who attended, the minor illness clinic of, the NHS GP surgery for their flu vaccination were invited by the local research lead. Minor illnesses are generally defined as a wide range of self-limiting conditions, normally managed through self-care (Wood, 2008). The local research leads informed potential cohorts about the study and asked them whether they were interested in taking part. The potential participants were assessed against the inclusion and exclusion criteria except for their BMI by the local research lead before sending them to me. Then the participant's height and weight were measured in the interview room by the student researcher to assess whether they met the inclusion standard for BMI. Participants who did not meet the inclusion and exclusion standards were thanked for their interest and offered a summary of the research findings upon their request. Participants who did meet the eligibility criteria and were still happy to take part were then asked to have a meeting on the same day or to arrange an interview date and time before they left the GP surgery.

**A pilot study**

The present study interview protocol was tested with the first two participants (case numbers 1 and 2). The same interview protocol was followed as agreed on for the actual interview. Therefore, the study recruitment process, consenting process, interview questions and the

recording device were tested to verify the clarity and accuracy of the whole process, as well as the appropriateness and effectiveness of the interview questions for the actual interview. The pilot test helped in determining the pace of asking questions and identifying the length of interview time needed for each participant. Finally, following the pilot test, all the interview questions were reviewed and refined.

**Data management**

For the present study, all field notes were scanned and saved as digital formats in a password-protected private laptop for the student researcher's personal use only. All participants were identified by numerical case numbers as discussed above. A digital tape recorder-Sanyo ICR-S25ORM was used to record all the interviews. All the recordings were transferred from the recording device to a personal laptop in the MP3 file. The audio files were transcribed in verbatim by the student researcher and saved in Microsoft Word format. Then the transcriptions are cleansed for any personal information to protect the anonymity of the participants. An independent researcher colleague double-checked the transcriptions against the audio tapes to verify their accuracy.

The data protection and retention comply with the University of West London's data protection policy. Once both audio and paper notes were converted to digital files, all paper documents and audio tapes were destroyed. All digital files were kept in a password-protected USB flash drive in a locked filing cabinet of the student researcher's house. Due to the pandemic, it was not possible to access the University computer, once the pandemic was over, data was saved in a password-protected University computer. The working files kept in the student researcher's laptop were free from any identifiable information and were only accessible to her. Data is stored in a USB flash drive and will be kept for up to 5 years after the end of the project, then the data will be disposed of appropriately.

## Trustworthiness and credibility

A prolonged relationship was developed with the study participants by establishing a positive, constructive interpersonal relationship during the recruitment process, before starting the actual interview, throughout the interview session and during the debriefing process. According to Lincoln and Guba (1985), a prolonged involvement with the interviewees can help the interviewer gain their faith and confidence and better understand their life philosophy or beliefs. Data were collected through several sources: interview recordings, field notes, transcripts, and reflective diary. The field notes and the reflective journal were shared with supervisors and an independent university researcher heard the recordings and read the transcripts to verify the transcriptions. The student researcher had an ongoing discussion with doctoral supervisors at every stage of the study. This enabled the student researcher to identify the gap in the process (such as the sequence of the questions) and helped to strengthen the study design. In addition, the study design was shared with my colleagues, peers, at different conferences, and the UWL Doctoral Research Committee for their feedback. The Doctoral Research Committee examined the study's methodology to verify the credibility of the methods applied in this study. Buman et al. (2010, pp. 225) argue that "The pragmatic approach to validity in science relies upon discourse and shared understanding of phenomena and ongoing reflections on the implications of research findings".

Most of the interview guide questions were adapted from ELSA survey self-completed questionnaires. The format of any questions that are adapted from ELSA was not changed. The open-ended questionnaires were discussed, checked, and proofread by doctoral supervisors. In addition, before finalising the questionnaire for a qualitative interview, a pilot study was undertaken based on a couple of respondents to validate the interview guide. The details of the pilot study were discussed in a previous section. The biases were openly and honestly discussed (student researcher's assumptions and orientation).

## DATA ANALYSIS

The narrative analysis permits insight into individual beliefs, judgements and social situations and allows the researcher to understand the stories in the study context. However, revealing the truth is not the purpose of narrative analysis, instead, researchers should recognise that there are possibilities of variation of truths (Holstein and Miller, 2007), that can be directed by participant's background and their socio-demographic or socio-economic conditions (Gergen, 1985). Among the four most common approaches of narrative analysis, structural analysis is the best suited, in which the primary focus is the way a story is built or presented (Holloway and Freshwater, 2007).

### Structural narrative analysis procedures

Each narrative is structured with the five common chapters in the chronological move towards the unmet care need. While every participant's life journey and stories are different, outlining a story with five common chapters provides a sequence. Each narrative started with the character introduction which was followed by life in terms of disability, health status and life satisfaction, existing care and support, concluding the interview with whether the interviewee wants to add anything more for their own care and support and finally, the results of unmet social care/support needs. The structural narrative analysis process involves analysing the transcription verbatim along with the digitalised field notes and reflective diary entries for one participant at a time. Each participant is presented separately to obtain an individual perception of the lived experience.

### Research findings

Research findings are discussed under three headings: participant background profile, a summary of the narrative discussion, and interpretation of the narrative. Specific cases are purposefully selected for narratives according to the study objective. After reading all the

transcripts several times, the interviews that answered the research question and provided the maximum information related to the study objective were chosen. The discussion of 'unmet care need' is framed around the framework (Figure: 1); a detailed description of the framework is presented above.

If the participants' reported 'unmet needs' are solely connected to the adverse effects of the COVID-19 pandemic, those participants are excluded from the narratives. Such as unemployment/financial hardship (for example, due to COVID company is closed […] *we both* (partner too) *lost our jobs* […] *worried about money*) or social isolation/ loneliness (for example, *I am a very social person, due to COVID* (pandemic), *I feel terrible* […] *wish to see my family and friends* […] *I had a very good social gathering before COVID*). In addition, two participants are excluded from the narratives as their 'unmet needs' are not related to the study objectives, for example, a high school teacher mentioned, *students taunt me* […] *due to my accent* […] *wish less racism and another interviewee mentioned that I feel I am not in the right place* […] *I don't feel socially safe* […] *I am here to make money*. In the narratives, symbols like: (.) represent pauses and […] represents omitted materials in the conversation, and italic words indicate the direct quotes from participants' talk, the exact way they told their narratives.

**Participant's profile**

Table 1 outlines each participant's demographic and background information and anthropometric height and weight measurements, calculated BMI, and assigned weight category. Table 2 provides the percentage prevalence of key background characteristics of the participants. Participants' demographical details, including their gender, age, ethnicity, marital status, or existing partner, are obtained (Table 1). Participants' weight category is assigned according to the WHO BMI classification, as discussed above.

## Table 1: Anonymised demographic details of participants

| Interviewees by case number | Age | Gender | Ethnicity | Marital status/existing partner | Height (m) | Weight (kg) | BMI (kg/m2) | Weight status (WHO categorised) |
|---|---|---|---|---|---|---|---|---|
| 1 | 78 | Male | British | Yes | 1.71 | 76 | 26 | Overweight |
| 2 | 50 | Female | Indian/ Mauritian | Yes | 1.65 | 115 | 42.4 | Class III obesity |
| 3 | 66 | Male | Bulgarian | Yes | 1.75 | 57 | 18.5 | Normal |
| 4 | 50 | Male | Russian | Yes | 1.70 | 60 | 23.2 | Normal |
| 5 | 60 | Male | British | Yes | 1.78 | 78 | 24.6 | Normal |
| 6 | 60 | Female | British | No | 1.54 | 56 | 23.6 | Normal |
| 7 | 57 | Male | Pakistani | No | 1.80 | 105 | 32.4 | Class I obesity |
| 8 | 58 | Male | Indian | Yes | 1.73 | 74 | 24.7 | Normal |
| 9 | 53 | Female | African | Yes | 1.60 | 102 | 39.8 | Class II obesity |
| 10 | 52 | Female | British | No | 1.60 | 102 | 39.8 | Class II obesity |
| 11 | 61 | Female | British | Yes | 1.55 | 105 | 43.7 | Class III obesity |
| 12 | 57 | Female | Indian | Yes | 1.52 | 79 | 26 | Overweight |
| 13 | 63 | Male | British | Yes | 1.75 | 87 | 28.4 | Overweight |
| 14 | 51 | Male | Spanish | Yes | 1.82 | 131 | 39.6 | Class II obesity |
| 15 | 75 | Male | Greek | Yes | 1.85 | 100 | 29.2 | Overweight |
| 16 | 58 | Female | British | Yes | 1.77 | 105 | 33.5 | Class I obesity |
| 17 | 59 | Male | Pakistani | Yes | 1.61 | 67 | 25.8 | Overweight |
| 18 | 62 | Female | British | Yes | 1.58 | 91 | 36.5 | Class II obesity |
| 19 | 66 | Male | British | Yes | 1.91 | 102 | 28 | Overweight |
| 20 | 52 | Female | British | Yes | 1.65 | 112 | 41.1 | Class III obesity |
| 21 | 73 | Male | British | No | 1.71 | 96 | 32.8 | Class I obesity |
| 22 | 55 | Female | British | Yes | 1.70 | 78 | 27 | Overweight |
| 23 | 54 | Female | British | Yes | 1.62 | 77 | 29.3 | Overweight |
| 24 | 69 | Male | British | Yes | 1.68 | 87 | 30.8 | Class I obesity |
| 25 | 60 | Male | British | No | 1.67 | 83 | 29.8 | Overweight |
| 26 | 69 | Male | British | No | 1.73 | 80 | 26.7 | Overweight |
| 27 | 52 | Female | British | Yes | 1.52 | 67 | 29 | Overweight |
| 28 | 63 | Female | Italian | No | 1.46 | 71 | 33.3 | Class I obesity |
| 29 | 56 | Female | British | No | 1.65 | 78 | 28.6 | Overweight |
| 30 | 57 | Male | British | Yes | 1.76 | 95 | 30.7 | Class I obesity |
| 31 | 74 | Male | British | Yes | 1.76 | 97 | 31.3 | Class I obesity |
| 32 | 53 | Female | British | Yes | 1.61 | 93 | 35.9 | Class II obesity |
| 33 | 55 | Male | British | Yes | 1.79 | 85 | 26.5 | Overweight |

## The main outcome of narrative analysis

A summary of 10 older adults (aged 50+ years), where each of them experienced an 'unmet care need', is presented below in Table 2. All names in the narratives are pseudonyms. The unmet care needs of each participant are in bold.

**Table 2: Narrative analysis findings**

| Case No. | Name and demographics | Narrative analysis |
|---|---|---|
| 2 | **Sunita (50 years, female, morbidly obese, positive cohabitation)** Sunita is a 50-year-old female, who lives with her husband. She is from an Indian/ Mauritian ethnic background and has morbid obesity (BMI= 42.4kg/m2). | <u>Life in terms of disability, health status and life satisfaction</u>: Sunita faces difficulty with several tasks in daily living, such as using the toilet, including getting up or down, preparing a hot meal, working around the house and garden, and climbing stairs due to her painful back and joints. However, she does not use any kind of mobility aids, and according to her, *I don't think I am that old.* She considers her health status as 'fair', however, does not consider that she has any type of chronic illness or mental impairment (health status):<br>*Generally, if you are good, you don't need medication, you don't […] you don't be in pain every time you wake up in the morning […] it just basically depends on the days, some days are good, but most days are like on a scale of 1 to 10 (higher indicates better health status), I would just call it like 5 and sometimes lower than this [...] it just depends on how you spend the night and […] I don't sleep […], so it just depends really.*<br>She feels safe in her own home and on a scale of 0 to 10 (higher indicates safer), she marks it 9. But she does not like being staying on her own (safety in the home):<br>*Well, if I remove the panic attack […], you know when you are ill (.) sometimes you feel you don't want to be alone […] because I was always surrounded by all my family […] especially if you are not feeling well. You start thinking of the worst (.) you know, so it just depends (.) so what I think about at home, if I am well surrounded, then it is a good 9, but if I am alone, I always panic (.) so I would say 4 (.) so depends on the time when he (husband) comes back home (.) I feel safe (.) I feel happy.*<br>Sunita considers herself 'unsatisfied' in terms of her overall life satisfaction, this is mainly due to the COVID-19 pandemic that has worsened the work situation and lets her be socially isolated. However, if not for the pandemic, she would have considered her life satisfaction as 'partially satisfied' (life satisfaction):<br>*Because of my health (.) fully satisfied would be someone, who does not need any support (.) you know they can rely on themselves, so they can do whatever they want in a way, but with my health and what is going on around (.) plus the pandemic makes worse.*<br><u>Existing care and support</u>: She has installed a speciality toilet (housing adaptation). She gets a "lot of support" from her husband, and she considers herself "very lucky". However, she feels her needs are 'usually met', though the amount of time her husband spends with her is not enough (care and support / unmet need): |

| | | |
|---|---|---|
| | | *He is the type of person who understands one word; you know if you say 5 words together, then the problem is (.) he doesn't have ears (giggles) […]. Still, he has to work […] because you know there is no flexibility from work; you know if you have support from a work (.) that's what the problem is at the moment […]. Still, they are not giving any support from his end to be able to provide the care that I need (.), so in a way, that's where the problem is (.), and you can't lose your job, especially of this time […], so he doesn't have the support needed to be able to support me […] sometimes I do need somebody to be around during the day (.) then either I have to go to a hospital or call a friend.*<br>Sunita is 'very satisfied' with how she presents herself in terms of her appearance and cleanliness. She is not so happy about the food and drink she gets at home (care and support):<br>*"he* (husband) *doesn't know how to cook […] because he always thinks that I need to lose weight and gives me healthy food (.) I can't really follow healthy diet".*<br>However, the way her husband treats her makes her feel excellent about herself.<br><u>Concluding the interview with whether the interviewee wants to add anything more for his/her own care and support:</u> Sunita wishes (unmet need): "*may be having someone to speak to (.) having someone to listen (.) and may be having a priority to access to a facility (.) you know when you are on your own and you don't have help (.) you need to call a place like hospital or book something […] for your own medication or something, you have to stay on the phone and at the same time you have to look after yourself (.)  so for these things […]  having a priority […] like giving a number […] and the third thing I believe that's more important is may be somebody like (.) more like a health care support or assistant just for cooking (.) not cooking, but food or […] may be just someone can come and see me (.) may be for an hour just to say hello (.) if I am not well, just to say (.) can I call an ambulance? (.) The person doesn't have to come with me but at least help me to get access to all the facilities […] may be a carer with compassion".*<br><u>The result on unmet social care/support need</u>s: From the above narrative, it is identified that Sunita feels vulnerable because her primary carer (her husband) is unable to spend the amount of time (**carer's time**) she feels is needed. **Loneliness** is one of the significant unmet needs that Sunita has experienced and sometimes that triggers panic attacks (**lack of emotional support). The lack of safety in her own home** is another concern likely associated with being alone in the house when her husband is at work and her family is not around. Moreover, Sunita **lacks the self-confidence to access existing social care services,** as she does not consider herself old enough to access certain types of support (mobility aids) and desires to have a more accessible support service. Furthermore, the foods she gets in everyday life do not match her wishes (**unmet want**). |

| | | |
|---|---|---|
| 27 | **Lucy (52 years, female, overweight, positive cohabitation)** Lucy is a 52-year-old female, who lives with her husband and is in full-time employment. Her children are all grown up and have moved away. Lucy is from a white British ethnic background and is overweight (BMI= 29 kg/m2). | <u>Life in terms of disability, health status and life satisfaction</u>: Lucy experiences several challenges for daily living, such as difficulty in walking, cutting up foods, getting in and out of bed, shopping for groceries, gets confused sometimes taking medications, working around the house and garden. However, she does not use any walking stick or any type of aid for moving. Lucy states her health status as 'fair', as she suffers from several conditions: osteoarthritis, erythromelalgia, and vitamin deficiency (the list is long) and, according to her, walking gives her pain (health status):<br>*pain is what I struggle with the most because that (exercise machine) all seem to cause pain […] most of the time I am fairly good (.) I am quite a sort of up bit and happy.*<br>Lucy feels very safe in her own home, even when her husband is not at home (safety in the home).<br>Lucy is 'fully satisfied' with her life overall (life satisfaction)<br>*[…] there are people far worse than me.*<br><u>Existing care and support</u>: Lucy's husband takes care of her besides being in full-time employment. Moreover, Lucy's husband is very supportive, as she says, *looks after me well*. Currently, he (husband) does not receive any care allowances. But taking care and support from her husband makes Lucy ‘partially satisfied’ (care and support):<br>*Sometimes I feel guilty (.) because I think he (Lucy's husband) was working hard as well, then he comes back home, he does the dinner […] washing, ironing, gardening (chuckles) he does everything. Although I have recently said to him (.) can you not do it? If you leave something for me (.) so that I don’t become a vegetable (stressed the word) (.) sitting on the sofa doing nothing!* (Long pause) *He says he does it because he doesn’t like to see me in pain*.<br>Moreover, she considers her needs are ‘usually met’ and the amount of time her husband spends with her is not enough, as she informs, I *wish it could be more* (care and support):<br>*I think I get by quite well (.) I think the only time I struggle mainly […] if I am going out and walking […] if I want to go to the shop […], I get put off […] I think you know (.) I got to walk from the car to the shop […] or going to the car park […] I have toyed with (.) may be applying for a badge thing (disability badge) then I get put off […] because then I think I can still walk, but (.) there are a lot of people out there that are more deserving of it […] I just think to myself […] at what point (.) do I give into that or (.) people are going to look at me (.) and think she doesn't need that.*<br>However, Lucy is 'fully satisfied' with her appearance, cleanliness, maintaining personal hygiene and the food and drinks she gets in everyday life.<br>But she considers that the help and support she receives from her husband makes her feel ‘poor’ (with a chuckle) about herself. She said that it makes her feel “*bit guilty, lazy, a burden (.) I know that doesn't make any sense […] yeah, I feel burden! (.) and then I think 'right (.) stop thinking about that, move onto the next.*<br><u>Concluding the interview with whether the interviewee wants to add anything more for his/her own care and support</u>: Lucy wishes (unmet need):<br>*I only need help with (.) maybe with that […] badge thing (disable badge), but that’s about it […] I am not going to start going down (.) using frames and walking sticks (.) because then, that just draws attention to it (.) and I think ‘no’* (chuckles). |

| | | |
|---|---|---|
| | | <u>The result on unmet social care/support need</u>s: From the above narrative, it is noted that Lucy perceives herself as a burden and feels guilty (**lack of emotional support**) for the support and care she receives from her husband; besides, she desires for more of his (husband) time (**carer's time**). Moreover, Lucy is reluctant to request help and **hesitant to accept having unmet needs,** as she compares herself with others around her and feels appreciative of what she has. "These attitudes led some participants to deny or minimise their needs for care and support, and/or to normalise challenging situations" (Dunatchik et al., 2017, p. 23). She puts a high value on her independence as an important part of her self-esteem. In addition, Lucy is afraid of being **socially discriminated** against. Therefore, she gives more importance to other's perceptions of herself than her needs. |
| 30 | **David (57 years, male, moderately obese, positive cohabitation)** David is a 57-year-old male, who lives with his wife. He is from a white British ethnic background and has moderate obesity (BMI= 30.7 kg/m2). | <u>Life in terms of disability, health status and life satisfaction</u>: David faces difficulty with almost all the activities of daily living. However, except with few activities that he considers himself capable of, such as communication, making phone calls and recognising physical danger. In addition, David uses several supporting aids that help him be independent, such as a walking stick, walker, wheelchair, and personal alarm for a fall. He suffers from several health conditions, including urinary incontinence, painful back due to lumbar decompression surgery. David considers his health status as 'poor' and is 'unsatisfied' in his overall life satisfaction (life satisfaction):<br>*I have depression […] I wasn't like this before (.) it's just too much suffering […] my wife changes my nappy (.) I can't help (.) makes me frustrated and depressed.*<br>David considers that he is not very safe in his own home and scored 5 (on a Likert scale of 0 to 10, where 10 is the safest). He also feels that *medication makes me drowsy (.) that I always feel weakness.*<br><u>Existing care and support</u>: David had several housing adaptations. Currently, he is getting disability allowance and housing allowance. He sees his wife as his primary carer. David is concerned that his wife does not get any government allowances because she is from abroad (care and support):<br>*We applied for LA (Local Authority) allowances (.) we meet all the criteria, still (.) it's a long fight […] we need to sort out her (wife) immigration first […] she deserves it (.) lawyer is fighting the case.* |

| | | |
|---|---|---|
| | | However, David is 'fully satisfied' with the care and support he receives from his wife in terms of his appearance, cleanliness and the food and drink he gets in everyday life. He says, *she is wonderful* (.) *she is always there*. The way his wife treats him makes him feel excellent about himself. But he considers his needs are 'sometimes met' (care and support):<br>*I wish to walk normally again (.) I start panicking with people […] don't like to mix with people* (agitated).<br><u>Concluding the interview with whether the interviewee wants to add anything more for his/her own care and support</u>: David expresses his worry about his wife's immigration. Since that would enable them to apply for the LA allowances. He concludes the interview with (unmet need):<br>*it would be helpful if I could have some economic support.*<br><u>The result on unmet social care/support need</u>s: From the above narrative, it is noted that David is frustrated with his current situation and feels guilty about taking help from his wife (**lack of emotional support**). David expresses his **social anxiety** (mixing with others), along with **financial hardship**. However, he yearns to walk again, and it is apparent that he misses socialising. David judges having a **lack of safety in his own home**. |
| 3 | **Ivan (66 years, male, normal weight, positive cohabitation)** Ivan is a married 67-year-old male who lives with his wife, son, and daughter in law. Ivan is from a white Bulgarian ethnic background and is normal weight (BMI= 18.5 kg/m2). | <u>Life in terms of disability, health status and life satisfaction</u>: Despite having a normal weight, Ivan encounters several challenges in daily living, such as difficulty in dressing, walking, getting in and out of bed, using the toilet and taking medication. He considers his health as 'fair'. Ivan is a bowel cancer patient, who had to go through a couple of surgeries but still suffers from severe pain and discomfort. Although he does not like to use any mobility aids and does not consider having any chronic illness or mental impairment (health status):<br>*Because of all this pain (.) I am getting severely; I am actually not getting any care […]. I was calling into the GP […] but was not able to get through (.) to get me some actual physical help.*<br><u>Existing care and support</u>: Ivan's wife is the main person who looks after him. And Ivan is happy for the time she stays with him. Moreover, Ivan is satisfied with the food and drink that he gets every day and happy with his appearance and cleanliness. However, taking support from his wife makes him upset, he is 'unsatisfied' with this and considers himself a burden. The way he is cared for or treated by his wife makes him feel 'poor' about himself (life experience/care and support):<br>*Obviously, my wife is caring (.) looks after me well […] but there are a lot of things that she is not enough (.) such as the medication, such as (.) knowing actually what the issue is with me (.) to manage it properly […] I need someone who has the knowledge to manage (.) someone who will stop me feeling embarrassed with my life (.) I was a very independent person […] in my culture, the husband should take care of his wife (.) not the other way round […] my wife is not well, she has osteoporosis.*<br>His needs are not met fully, mainly due to the pain, which marks it as 'sometimes met'. According to him, *I need help (.) with my doctor to understand my problem* (care and support).<br><u>Concluding the interview with whether the interviewee wants to add anything more for his/her own care and support</u>: Ivan shows concern about his wife's health and wellbeing and mentions that she is helpless. As Ivan expresses his expectation (unmet need):<br>*I guess I need to have some sort of help from the government for extra money or fund (.) maybe it could help me (.) because it costs a lot for a good healthy diet.* |

| | | |
|---|---|---|
| | | <u>The result on unmet social care/support need</u>s: From the above narrative, it is noted that Ivan is embarrassed by taking the support and care from his wife, who is already unwell and considers himself a burden **(lack of emotional support)**. In addition, Ivan feels vulnerable and shows concern that his wife lacks the necessary skills to provide him proper support (**lack of carer's knowledge**) besides his **financial hardship**. Moreover, his expressed needs are sub-optimally satisfied, and he is insecure about his existing unpaid care. Finally, he is having **lack self-confidence in obtaining existing social care services.** |
| 10 | **Lisa (52 years, female, severely obese, positive coresidents)** Lisa is a 52-year-old lady who is recently divorced and lives with her daughter. Lisa is from a white British ethnic background and has severe obesity (BMI= 39.8 kg/m2). | <u>Life in terms of disability, health status and life satisfaction</u>: Lisa is entirely self-sufficient and capable of doing all the tasks for daily living independently. Lisa marks her health as 'good'. However, Lisa lists several health problems, including carcinogenic neck lump and ongoing kidney problem with 4% kidney function restored (health status / life experience):<br>*I just had a nasty divorce (.) was trying to cope up (.) with the situation (.) you know it was hard, my daughter is very supportive (.) but dealing with the bereavement of my parents is harder […] I started having mental health problem (.) anxiety, depression […] was getting better with counselling (.) but had to stop.*<br>Lisa feels very safe in her own home since she is there for 23 years (safety in the home).<br>Therefore, she reveals that she is 'partially satisfied' in her life, mainly due to bereavement and financial problems (life satisfaction):<br>*it's very difficult being on your own (.) hard to deal with the loneliness […] you know, at least someone to speak (.) when I am in need (.) in the middle of the night.*<br><u>Existing care and support:</u> Lisa started having counselling service for the last 3 months, and she felt that her condition was improving. However, she had to pay for the service independently, and it was very stressful for her. As Lisa mentions, *I stopped now because I couldn't afford it.* Lisa is 'fully satisfied' with her appearance and the food and drink she has in everyday life. But her needs are not fully met (care and support):<br>*I have a good circle of friends (.) good family (.) still awful sometimes staying on my own (.) lack of emotional support […] especially at the weekends (.) Sundays (.) feel like to have someone.*<br><u>Concluding the interview with whether the interviewee wants to add anything more for his/her own care and support</u>: Lisa expresses her worry about her financial situation. Lisa experiences that there is a sort of gap between becoming single also reduces your income, because she does not have a partner who is financially supporting each other. Finally, she wants more accessible support or care (unmet need).<br><u>The result on unmet social care/support need</u>s: From the above narrative, it is noted that Lisa conveys her void of **loneliness** and **lack of emotional support** throughout her interview besides having **financial hardship**. She desires to have **more accessible support service**. Moreover, the privately paid counselling therapy was helping her but had to stop due to her financial hardship. In addition, she has a **lack of self-confidence obtaining existing social care services.** |
| 32 | **Alison (53 years, female, severely obese, positive cohabitation)** | <u>Life in terms of disability, health status and life satisfaction</u>: Alison faces several difficulties with the activities of daily living. Moreover, she is dependent on a walking stick; and sometimes she uses wheelchair and elbow crutches. In addition, she has a couple of health issues, mostly related to problems with her back and joints due to arthritis and |

| | | |
|---|---|---|
| | Alison is a 53-year-old lady, and lives with her partner. She is from a white British ethnic background and has severe obesity (BMI= 35.9 kg/m2). | osteoporosis. A few of her health conditions are long standing (health status).<br>Alison marks 7 out of 10 (on a Likert scale of 0 to 10, where 10 is the best) regarding safety in her own home (safety in the home):<br>*Mainly due to the stairs in the house (.) sometimes I feel dizzy (.) weak (.) sometimes I feel like (.) I might fall down one day.*<br>Alison is 'partially satisfied' with her life overall (life satisfaction):<br>*it varies and depends on the day (.) I have good days and bad days (.) but mostly bad (.) because lack of mobility makes me not so satisfied (.) I get depressed and frustrated (.) for being in pain (.) for taking so many pills (.) due to COVID, can't even go for swimming or other exercises.*<br><u>Existing care and support:</u> Alison has a couple of housing adaptations like raised toilet and bath seat. However, she wants the bath to be taken out, as the shower will be more accessible. Alison had a few sessions of physio exercises that she did not have to pay. But she regrets that due to the pandemic, her physio sessions are cancelled. Alison's partner is her primary carer. Currently, Alison is receiving PEEP (Personal emergency evacuation plan) and reduced council tax. And her partner has just applied for a carer's allowance. The care and support she receives from her partner make her feel guilty (care and support / unmet need):<br>*he is good (.) it's been a roller-coaster (.), but it's pretty good now […] sometimes struggle to make him realise the need is.*<br>She marks herself 'partially satisfied' in terms of the care and support she receives from her partner. However, Alison informs that she is happy about her partner's time and states that *sometimes I like to spend time on my own*. But she regards that her needs as 'hardly ever met'. According to her, *I don't feel the way I was before (.) can't do socialising*.<br>The way Alison's partner cares and looks after her makes her feel 'very good' about herself. However, the way others treat her makes her feel 'fair' about herself. As she conveys her feelings,<br>*He (partner) does try to understand […] he is getting older (.) he has medical needs too […] sometimes I feel like a burden […] a few months back, he fell ill (.) I was calm; it was difficult for me […] children came to rescue and looked after me […] family and friends don't understand me fully (.) my condition.*<br>She is 'fully satisfied' with her appearance and cleanliness. However, due to her food intolerance, it is difficult for her to get the right food.<br><u>Concluding the interview with whether the interviewee wants to add anything more for his/her own care and support</u>: Alison conveys her desire to do more exercise and considers that few more housing adaptations are needed to make her life easier to live (unmet need):<br>*The realisation from people or everyone (.) that I have good and bad days […] I want people's empathetic understanding towards me (.) not to decide by seeing my appearance and condition […] I don't want to be disabled (.) treated as disabled (.) trying to have a normal life.*<br><u>The result on unmet social care/support need</u>s: From the above narrative, it is noted that Alison feels guilty for receiving care from her partner and perceives herself as a burden (**lack of emotional support)**. She faces challenges in socialising **(**lack of socialisation) with others. In addition, she gets a sense of **social discrimination** while tries to mix with others. Moreover, Alison feels vulnerable due to a **lack of housing adaptations** and **lack of safety in her own house** due to the precarious nature of her existing unpaid care. In addition, Alison puts a high value on her independence to protect her self-esteem and misses doing her hobbies (swimming, exercise, physiotherapy) due to the pandemic. |

| | | |
|---|---|---|
| 7 | **Ali (57 years, male, moderately obese, on his own)**<br>Ali is a 57-year-old widow and lives on his own. He is from a British Pakistani ethnic background and has moderate obesity (BMI= 32.4 kg/m2). | <u>Life in terms of disability, health status and life satisfaction</u>: Ali faces a problem using the toilet and using a bath as part of the difficulties in daily living. Although he does not use any type of aids to make his moving or daily living easier. Ali considers his general health is 'fair', mainly due to back pain and joint pain. However, he does not regard having any chronic illness (health status.<br>Ali assesses his safety score 5 out of 10 (on a Likert scale of 0 to 10, where 10 is the best), living in his own house (safety in the home). Moreover, he interprets his overall life satisfaction as 'partially satisfied' (life satisfaction).<br>As Ali appraises his health and wellbeing:<br>*When I go to the bath, I have to be very careful (.) I couldn't stand for long […] I mean to say, there is not enough support to hold onto something […] I have slipped a couple of times (.) no walking shower (.) I do everything on my own […] there is no support (.) one lady came to visit me and said, she is going to write to my county or borough […] she wrote, but nothing happened […] I don't have any other health problems, but the back pain is the worst of all […] when I don't feel good, I try not to go out […] I were living in this flat (Local Authority housing) for the last 12 years […] I feel, people, come in my absence […] building entrance has a problem with lock […] lack of security […] because of my back pain, I lost my job (.) then all the problems started.*<br><u>Existing care and support</u>: According to Ali, currently, he is not receiving any care or support, and his needs are 'hardly ever met'. Although he is 'fully satisfied' with his cleanliness and appearance. However, the foods and drink he gets in everyday life are not enough for him, making him 'unsatisfied' (care and support / unmet need):<br>*I applied for the job seeker's allowances a few months back (.) but they refused (.) by saying that 'you didn't pay for the taxes, so you can't apply for this' […] if the employer doesn't give me enough shifts that I could pay the tax […] they only give me two days jobs […] it's not my fault [...] I pay for my national insurance number […] the situation is frustrating […] as I don’t have any choice, managing as much as I can […] basically compromising.*<br><u>Concluding the interview with whether the interviewee wants to add anything more for his/her own care and support</u>: Ali expresses his desire to have a separate apartment with a walk-in shower (unmet need):<br>*if possible, to have proper support with few housing adaptations [...] as I live on my own […] help with daily living, such as the washing machine is in the distance […] even picking up something from the floor, makes me worried.*<br><u>The result on unmet social care/support need</u>s: From the above narrative, it is noted that he is frustrated about his circumstances (**lack of emotional support**) besides having **financial hardships**. Moreover, Ali feels vulnerable due to **a lack of housing adaptations** and **safety in his own house.** His expressed needs are sub-optimally satisfied, as he does not qualify to access social care support (service-based). |
| 19 | **Andrew (66 years, male, overweight, positive cohabitation and coresidents)** | <u>Life in terms of disability, health status and life satisfaction</u>: Andrew encounters several challenges of daily living, such as using a map, recognising physical danger, preparing a hot meal, shopping for groceries, taking medication, managing money and sometimes with communication and making phone calls. Andrew gets confused and cannot remember everything. He does not like to use a walking stick, although he falls over a lot. And all these started after Andrew had a |

| | | |
|---|---|---|
| | Andrew is a 66-year-old man and lives with his wife and daughter. He is from a white British ethnic background and is overweight (BMI= 28 kg/m2). | stroke in 2012, but Andrew considers his general health is good (Health status).<br>However, he does not feel very safe in his own home. And overall, Andrew perceives his life satisfaction as *very poor* (unsatisfied) (safety in the home). As Andrew examines his health and wellbeing:<br>*I can't remember what I am doing (.) sometimes I try to do something, I get confused (.) suppose I am making a cup of coffee (.) I can't remember how many spoonsful of coffee I have added (.) I just keep on adding […], then when I drink it, it tastes awful, and I get confused (.) I am sorry that I am explaining this […] I know I fall over a lot (.), but I keep saying to myself, I never ever going to use a wheelchair or anything like that (.) I probably sound stupid […] the days I feel unwell, I like to go out (.) I know it can be dangerous, but I have to keep trying (.) if I don't feel right, I just sit down a bit […] The days I am good, try to stay at home […] I get really upset.*<br>Existing care and support: Andrew's wife and daughter look after him. And Andrew feels fully satisfied having the care and support from them. Andrew is happy about the time his wife and daughter spend with him. However, Andrew interprets that his needs are usually met. He is not so satisfied with his appearance and cleanliness and states, *I accept myself the way I am now.* However, Andrew is 'fully satisfied' with the foods and drink he gets in everyday life, and the care and support makes him feel excellent about himself (care and support / unmet need):<br>*They* (wife and daughter) *are wonderful (.) but I can't remember if I get any other supports! […] everyone loves me now, obviously, that's the wonderful part of it (.) but most of the time (.) in my head (.) I get very upset to myself […] I wait for my wife or daughter to come back home from work (for food) […] I do try on my own, all I tend to take is (.) a Pizza (.), and I don't like it, but that takes 12 mins to microwave (.). If I don't have it, then I don't know what I am dealing with […] I look at egg (.) sometimes I feel like to have it (.), but I can't remember what to do with it (.) then I get confused […] So even I don't like Pizza, I still have it […] at least I got something to eat.*<br>Concluding the interview with whether the interviewee wants to add anything more for his/her own care and support: Andrew expresses that he cannot follow a conversation if in a group but feels like contributing during a one-to-one conversation. Andrews accepts that it is difficult for someone to look after him, but thankful for his wonderful family, who is always ready to serve him. Andrew loves mixing with people and loves to share his experiences. Although he cannot remember everything, he loves helping other people in need (unmet need).<br>The result on unmet social care/support needs: From the above narrative, it is noted that Andrew has several existing needs in terms of ADLs and IADLs, but lack of support with everyday activities necessitates. Although he is having some supports from his family (informal carers), he is somehow neglected (**have inadequate existing care**) due to their (carer's) busy schedules (**lack of carer's time**). Moreover, Andrew tries to compromise with his present situation and expresses his frustration (**lack of emotional support).** In addition, he is vulnerable in his own home in terms of his foods and drinks, staying on his own for a long time (**lack of safety in his own house**). In addition, Andrew puts a high value on his independence to protect his self-esteem and misses being independent. And he feels his self-worth by getting the chance to help others. |

| | | |
|---|---|---|
| 21 | **Kevin (73 years, male, moderately obese, on his own)**<br>Kevin is a 73-year-old widow and lives on his own. He is from a white British ethnic background and has moderate obesity (BMI= 32.8 kg/m2). | <u>Life in terms of disability, health status and life satisfaction</u>: Despite having a heavy-weight, Kevin is entirely self-sufficient and capable of doing all the tasks for daily living independently and considers his general health 'good'. Although he had his double knee replacement 6 years ago and existing arthritis with painful fingers (health status).<br>He feels reasonably safe in his own house and scores it 9 out of 10 (where 10 is the safest), and fully satisfied with his life overall (safety in the home).<br>*Since then, although I don't have any problem* (following knee replacement*) […] I find that I can't run (.) and I like to say, I can't kneel down (.) and also (.) once I used to do my own decorating, I would kneel down and paint the skirting and all that (.) but now I can't do anymore (.) that's the only drawback […] I feel quite safe, but always that possibility that something happens (.) in my back of mind (.) as no one is there.*<br><u>Existing care and support</u>: Kevin does not receive any form of social care support. He is 'fully satisfied' with his appearance and the foods and drinks he has in everyday life; besides, the way everyone treats him makes him feel 'very good' about himself (not excellent). However, all his needs are not met fully, as he mentions 'usually met' (care and support / unmet need):<br>*Obviously, with this circumstance* (COVID-19 pandemic) *now, I receive plenty of offers from my neighbours […] if I need any shopping or anything! (.) apart from that, I do all myself […] my wife passed away 6 years ago […] couple of years later, I applied for a part-time job (.) taking special need children to and from school […] so that's get me out of the house […] obviously, interacting with people keeps me busy […] obviously, during the evening, I am on my own […] that's why I do this driving job […] that keeps me busy and (.) my mind from going crazy […] my sons are away (.) they are busy in their own life (.) I always keep in touch with my family members […] obviously, before my wife passed away, I mean I walked in from work, and my meals were on the table […] I have to do all that myself now! (.) and I decided, I don't want to live on fast food […] so, I try to make a balanced diet, something different every day.*<br><u>Concluding the interview with whether the interviewee wants to add anything more for his/her own care and support</u>: Kevin ends the conversation by saying that he is reasonably *content* and happy to live independently (unmet needs):<br>*Don't need anything more […] like I said, there is always someone there, at the other end of the telephone […] I am happy with my life.*<br><u>The result on unmet social care/support need</u>s: Kevin misses out on doing his old hobby (decorating) and his run, as his health condition does not permit that anymore (**lack of emotional support**). Moreover, living alone makes him paranoid about his situation that someday something might get wrong (**lack of emotional support**). In addition, Kevin is reluctant to request help and **hesitant to accept having unmet needs.** But Kevin feels coded by getting the chance to do part-time work and puts a high value on his independence. However, Kevin admits that he does this job mainly to protect his mental health by being able to engage his mind and being able to socialise with others (*obviously, during the evening, I am on my own* […] *that's why I do this driving job* […] *that keeps me busy and* (.) *my mind from going crazy*) (to avoid being **lonely** and **socially isolated**). |

| | | |
|---|---|---|
| 14 | **Daniel (51 years, male, severely obese, positive cohabitation)**<br>Daniel is a 51-year-old male and lives with his wife. He is from a white Spanish ethnic background and has severe obesity (BMI= 39.6 kg/m2). | <u>Life in terms of disability, health status and life satisfaction</u>: Despite having a heavy-weight, Daniel is entirely self-sufficient and capable of doing all the tasks for daily living independently and considers his general health very good (health status).<br>Moreover, Daniel feels very safe in his own house. Although he has gout with very painful left foot and knees (safety in the home).<br>*Somehow my energy is drained (.) I have sleep apnoea (.) I think the actual problem is there […] I am doing lots of exercises, but not lost enough weight […] sometimes I stop walking or exercise, because of gout […] I feel frustrated […] not losing weight regardless of doing exercise.*<br><u>Existing care and support</u>: Daniel feels 'fully satisfied' with the support he receives from his wife. Besides, Daniel is pleased with his wife's time with him and 'fully satisfied' with his appearance and the food and drinks he has in everyday life. Moreover, Daniel judges that all his needs are 'fully met' and feels 'very good' about himself how his wife and others treat him. As he examines his care needs (care and support / unmet need):<br>*My wife is always there to help (.) but I try not to ask too much, try to be independent […] sometimes our opinion differs […] I like to do things in a particular way (.) want to listen to a lot of music (.), but she doesn't like […] it's too noisy for her and (.) I have to always use a headphone […], not everything is perfect, there is always a room for improvement […] looking the way some people live (.) I live comfortably, but if there is money (.) I could live more comfortably.*<br><u>Concluding the interview with whether the interviewee wants to add anything more for his/her own care and support</u>: Daniel ends the interview by thanking me for giving him the opportunity and time to express his views and says *I hope the study helps to get things right*.<br><u>The result on unmet social care/support need</u>s: Daniel wishes to be fitter and lose weight but gets disappointed and frustrated (**lack of emotional support**) for not achieving weight loss as he desires. Besides that, although his wife is supportive, sometimes the difference of opinions makes him annoyed (**lack of emotional support**). Moreover, Daniel is reluctant to request help and **hesitant to accept having unmet needs.** Financially Daniel is stable, but he feels upset while comparing himself with others (**lack of emotional support**). |

According to the conceptual framework (Figure 1), all the participants in the narratives in Table 2 either had 'unexpressed needs' (D) or 'expressed needs that were sub-optimally satisfied' (C2). The ‘unexpressed needs’, where participants either had care needs in terms of ADLs, IADLs, and mobility but did not perceive their difficulties (case numbers- 19, 14) or participants were reluctant to request help and support (case numbers- 19, 27, 21, 14, 32). The ‘expressed needs that were sub-optimally satisfied', where participants either not qualified to meet the eligibility criteria to access the local authority social care support (case number- 10, 7, 14) or had

inadequate existing social care (case number- 2, 27, 30, 32, 19) and support. Some participants also had both unexpressed needs and expressed sub-optimally satisfying needs (case numbers- 27, 14, 32, 19).

The participants with impaired mobility or problems in daily living faced the following challenges of unmet needs: ill health, loneliness, lack of socialisation, lack of emotional support (felt frustrated, depressed, burdened, guilty, embarrassed and anxious), felt vulnerable due to lack of carers' time (unpaid carers), lack of carers' knowledge, lack of housing adaptations, lack of support with everyday activities, lack of self-confidence obtaining existing social care services, lack of safety in one's own house and lack of financial aid (individual raised).

From the above narratives, it is discerned that most of the participants mentioned their painful back and joints resulting from different types of arthritis (case number- 2, 27, 53, 57), as the main reason for their disability, or the pain due to cancer, kidney dysfunction and previous surgery.

Participants with increasing levels of obesity reported most of the unmet needs related to the lack of emotional support due to stress and frustration, disappointment, lack of social contact, feeling of social discrimination, feeling of being a burden, lack of housing adaptations, lack of safety, boredom or being unable to continue one's hobbies, lack of easily accessible support system. Whereas the normal weight and overweight participants (case numbers- 3 and 19) had unmet needs that were mainly related to the gaps in service provision, such as lack of carer's time and knowledge.

The above narratives also reveal that participant's past health conditions (case numbers- 21, 14); their difficulties in daily living and mobility (case numbers- 3, 7, 19, 32, 30, 27, 2) or a combination of the above two and significant life events, like bereavement, divorce (case number- 10) generate unmet needs.

The above narratives show that older adults living with their spouse/ partner and/or children are getting some help and care than those living on their own.

The above narratives also show how the sense of frustration, social discrimination, lack of life purpose, depression, being a burden, guilt, embarrassment, anxiety, boredom, and being unable to do things they used to do diminish participants' self-worth and self-confidence. In addition, the narratives reveal that despite having a sound support system in terms of money, housing adaptations, mobility aids and unpaid care support (case number- 27) or not having any difficulty in daily living (case number- 21, 14) the older adult participants can still experience low self-esteem.

From the above narratives, it is noted that loneliness and social isolation are more critical issues for many older adults (case number- 2, 10, 32, 21) than actual physical needs regardless of their disability and health status.

Some older adults were also afraid of being socially discriminated against (case numbers- 27, 32), socially judged (case numbers- 27, 32) and have social anxiety (case numbers- 30) or panicked while mixing with other factors that emerged from the narratives which in turn seems to lead to further social isolation or lack of socialisation and then poor wellbeing.

For many older adults taking care and support from their partner/ spouse (for some cases, taking care and support from the wife is not culturally accepted, for example, case number 3), itself is one of the major predisposing factors of having poor wellbeing (case number- 27, 30, 32). It is seen from the above narratives that some older adults with fair/poor life satisfaction are more prone to have poor wellbeing and feel less good about themselves, perhaps resulting in several unmet needs regardless of the care and support they receive (case numbers- 2, 30, 10, 32, 7).

On the other hand, Daniel (case number 14) gets frustrated with his life for not losing the desired weight. Although he is hesitant to accept having unmet needs, 'lack of emotional support’ is one of the unmet needs emerging from his conversation. In addition, his tiredness

and lack of energy due to sleep apnoea resulted from his high BMI (Rössner, 2001; LGA, 2020).

Some older adults are more satisfied in their lives overall, despite their unstable care and support system. Those are the people who have hobbies and recreational activities (case number- 32, although due to the pandemic, Alison cannot continue and that makes her upset), they have an opportunity to help others (case numbers- 19, 21), or they take up part-time /full-time work (case number- 21, 2, although Sunita is currently unsatisfied with her life, as due to the pandemic Sunita is out of work and socially isolated), having family and friends to talk (even though via phone call: case number- 21) and having a positive cohabitation relationship (case number-14).

It is also noted that financial hardship is another unmet need that older adults are having. For example, Ali (case number 3) was in full-time employment, but he lost his job due to his painful back, and joints resulting from his obesity. However, older adults who put a high value on their independence to protect their self-esteem and hold a positive outlook are deemed to have higher wellbeing regardless of their increasing weight and also seem to have fewer unmet care needs than others (case number- 21, 27). The above narratives, also reveal that in many cases, minimal support like few adaptations or aids or just providing a counselling service (case number- 10) and/or providing a social network may help older adults to meet their needs.

## DISCUSSION

The narrative analyses of the qualitative semi-structured interview indicate that participants face challenges in their everyday lives regardless of their weight categories. Moreover, the stories reveal that older adults with disabilities concerning difficulties in either ADLs, IADLs and/or mobilities struggle to meet a range of compound needs than other older adult participants. The findings are supported by Age UK (2019) that found living with a single 'need' can be as stressful as living with compound needs and that if they remain unmet, then

individuals are often struggling with a combination of unmet needs Several past studies have found that various types of arthritis, cancer, and kidney disease can be directly or indirectly associated with individuals' high BMI level (Sach et al., 2006; Nizalova et al., 2018; Leal Neto et al., 2016). This is also reflected in the narrative stories of the study participants that the main reason for their disability is either due to their painful back and joints or the pain due to cancer, kidney dysfunction and previous surgery.

Moreover, the narratives of the present study explore that loneliness and social isolation are critical issues for many older adults regardless of their disability and health status. Yet loneliness is a key predictor of poor self-esteem and lack of self-confidence (Dykstra, 2009). A longitudinal study on English older adults by Shankar et al. (2015) explored that loneliness and social isolation are associated with an individual's poor wellbeing. These findings are also in line with a NatCen study by Dunatchik et al. (2017, p. 6) from a primary interview dataset of older adults that “older people raised unmet need for social contact and mobility as being as important, if not more important as meeting basic needs of daily living". Furthermore, several studies support the finding that high BMI increases the risks/likelihood of loneliness, social discrimination, being socially judged and social anxiety (Day et al., 2018; Jung and Luck-Sikorski, 2019; LGA, 2020).

Furthermore, it is noted from the interview narratives that some older adults are more satisfied in their lives overall, despite their unstable care and support system. Age UK (2019, p. 20) states that “wellbeing for older people is multi-faceted and includes health, care and support, money, housing and social contact. It follows that people living with disadvantage in these areas, and even with a single need, are more likely to have lower wellbeing". A longitudinal study on the secondary ELSA dataset by Dunatchik et al. (2019) found that older adults’ level of wellbeing is not a significant predictor of their future unmet care needs. Another report by Dunatchik et al. (2017, p. 66) on a primary interview dataset of older adults showed that there

are some areas of unmet care need that have a stronger association with wellbeing than others, these are "lack of mobility and isolation and lack of access to hobbies and interests and the associated loss of independence”. However, both studies considered the study participants included older adults that may be underweight and malnourished and so may not wholly apply to overweight and older adults with obesity. At the same time, the study by Dunatchik et al. (2017) found that an easily accessible helpline or community centres can be an intervention that reduces the risk of negative wellbeing. This is in line with this present study’s findings that many older adults are frustrated and upset due to a lack of easily accessible support or helpline (case number- 2, 10).

Financial hardship is found to be another crucial issue for many older adults in the present study since there is an association between obesity and unemployment (LGA, 2020), and older adults experiencing major transitional phases of life, like retirement (Han et al., 2011). As such, a cross-sectional study by Conklin et al. (2013) established obesity demands financial hardship regardless of social class, education, and housing tenure. However, older adults who put a high value on their independence to protect their self-esteem and hold a positive outlook are deemed to have higher wellbeing. Although, this finding needs to be treated with caution as according to Dunatchik et al. (2017, p. 6), "managing to cope, but with impacts on exhaustion and pain, or by limiting expectations is an indication of unmet need".

It is, however, important to state that qualitative interviews reveal people's perspectives to see their lives rather than quantify the number of people holding those outlooks (Dunatchik et al., 2017).

## CONCLUSION

Insight into the unmet care needs of overweight and older adults with obesity compared to normal-weight older adults in England is being presented uniquely as part of the primary study question. Additionally, it briefly presents those central theories that help the present study

findings to be grounded. In contrast to past studies, the present study incorporates the more recent holistic viewpoint regarding overweight and obesity in older adults. Furthermore, it is noted that poor health and wellbeing increase several unmet needs among older adults. As such, the above narratives reveal that participants with obesity are more vulnerable to having health inequalities due to a lack of emotional support, resulting from a feeling of being a burden, disappointment, lack of social contact, feeling of social discrimination, lack of housing adaptations, feeling of lack of safety, boredom or being unable to continue one's hobbies, stress and frustration, lack of easily accessible support system, than their normal weight and overweight counterparts. Subsequently, this lack of emotional support perhaps leads them towards adopting poor health behaviour. Moreover, it was found that the addition of minimal public social care support could help older adults meet these unmet needs in many cases and improve wellbeing. The study outcomes will enable policymakers and the health and social care providers to have a new perspective and insight into the needs of overweight and older adults with obesity and how they can design an obesity management service for overweight and older adults with obesity in England that considers both health and social care needs in a more holistic way. Furthermore, social care should empower and enable individuals to live as independently as possible focusing on their abilities rather than disabilities.

## REFERENCES


Abdelaal, M., le Roux, C.W. and Docherty, N.G. (2017) 'Morbidity and mortality associated with obesity'. *Annals of Translational Medicine*, 5(7), pp.161–161. Available at: https://www.ncbi.nlm.nih.gov/pmc/articles/PMC5401682/ (Accessed: 10 June 2020).

Age UK (2019) *Estimating need in older people Findings for England*. Available at: https://www.ageuk.org.uk/globalassets/age-uk/documents/reports-and-publications/reports-and-briefings/active-communities/id204303-estimating-needs-report.pdf. (Accessed: 13 November 2019).

Age UK (2014) *Life Course Approach to Healthy Ageing*. Available at: https://www.closer.ac.uk/wp-content/uploads/Halcyon_A-life-course-approach-to-healthy-ageing-brochure.pdf (Accessed 2 May 2021).

Alexandre, T. da S., Scholes, S., Ferreira Santos, J.L., Duarte, Y.A. de O. and de Oliveira, C. (2018) 'The combination of dynapenia and abdominal obesity as a risk factor for worse trajectories of IADL disability among older adults.' *Clinical Nutrition*, 37(6), pp.2045–2053. doi: 10.1016/j.clnu.2017.09.018.

Bandura, A. (2004) 'Health promotion by social cognitive means.' *Health education & behavior: the official publication of the Society for Public Health Education*, 31(2), pp. 143–164. https://doi.org/10.1177/1090198104263660.

Batterham, R. (2020) *Health inequalities and obesity*. Royal college of physician. London. Available at: https://www.rcplondon.ac.uk/news/health-inequalities-and-obesity. (Accessed: 2 February 2021).

Bradshaw, J.R. (2013) *Jonathan Bradshaw on Social Policy: Selected Writings 1972-2011*. University of York, York, pp. 345. Available at: http://eprints.whiterose.ac.uk/112541/.

Brimblecombe, N., Pickard, L., King, D. and Knapp, M. (2016) 'Perceptions of unmet need for community social care services in England. A comparison of working carers and the people they care for.' *Health and Social Care in the Community*, 25(2), pp. 435-446. doi: https://doi.org/10.1111/hsc.12323.

Buman, M.P., Daphna Yasova, L. and Giacobbi, P.R. (2010) 'Descriptive and narrative reports of barriers and motivators to physical activity in sedentary older adults.' *Psychology of Sport and Exercise*, 11(3), pp.223–230. doi: 10.1016/j.psychsport.2010.02.002.

Commission for Social Care Inspection (CSCI) (2008) *Cutting the Cake Fairly: CSCI review of eligibility criteria for social care*. CSCI. Available at: http://www.cpa.org.uk/cpa/cutting_the_cake_fairly.pdf (Accessed: 24 November 2018).

Conklin, A.I., Forouhi, N.G., Suhrcke, M., Surtees, P., Wareham, N.J. and Monsivais, P. (2013) 'Socio-economic status, financial hardship and measured obesity in older adults: a cross-sectional study of the EPIC-Norfolk cohort'. *BioMed Central Public Health*, 13(1). Available at: http://www.biomedcentral.com/1471-2458/13/1039. (Accessed: 29 April 2018).

Copley, V.R., Cavill, N., Wolstenholme, J., Fordham, R. and Rutter, H. (2017) 'Estimating the variation in need for community-based social care by body mass index in England and

associated cost: population-based cross-sectional study'. *BioMed Central Public Health*, 17(1). doi 10.1186/s12889-017-4665-1.

Davis, M.M. (1955) *Medical care for tomorrow*. Harper, New York.

Day, F.R., Ong, K.K. and Perry, J.R.B. (2018) 'Elucidating the genetic basis of social interaction and isolation'. *Nature Communications*, 9(1). Available at: https://www.nature.com/articles/s41467-018-04930-1 (Accessed: 28 April 2019).

Degener, T. (2014) *A human rights model of disability*. Available at: https://www.researchgate.net/publication/283713863_A_human_rights_model_of_disability. (Accessed: 17 October 2018).

Department of Health (DOH) (2014) *Care and Support Statutory Guidance*. Issued under the Care Act 2014. Available at: https://www.bl.uk/collection-items/care-and-support-statutory-guidance-issued-under-the-care-act-2014-1. (Accessed: 15 December 2019).

Department of Health and Social Care (2021) *Care and support statutory guidance: Using the Care Act guidance.* Available: https://www.gov.uk/government/publications/care-act-statutory-guidance/care-and-support-statutory-guidance#using-the-care-act-guidance. (Accessed: 28 April 2021).

Djalalinia, S., Qorbani, M., Peykari, N. and Kelishadi, R. (2015) 'Health impacts of obesity'. *Pakistan Journal of Medical Sciences*, 31(1), pp. 239-242. doi: http://dx.doi.org/10.12669/pjms.311.7033.

DeJonckheere, M. and Vaughn, L.M. (2019) 'Semistructured Interviewing in Primary Care research: a Balance of Relationship and Rigour'. *Family Medicine and Community Health,* 7(2), pp.1-7. doi:10.1136/fmch-2018-000057.

Dunatchik, A., Icardi, R. and Blake, M. (2019) 'Predicting Unmet Need for Social Care.' *Journal of Long-Term Care*, pp.194. http://doi.org/10.31389/jltc.33.

Dunatchik, A, Icardi, R, Roberts, C and Blake, M. (2017) 'Predicting unmet social care needs and links to well-being: Findings from the secondary analysis.' *NatCen Social Research and Ipsos MORI*: London. Available at: https://www.ipsos.com/ipsos-mori/en-uk/ unmet-social-care-needs-and-well-being (Accessed: 10 December 2020).

Dykstra, P.A. (2009) 'Older adult loneliness: myths and realities.' *European Journal of Ageing*. 6(2), pp.91–100. Available at: https://www.ncbi.nlm.nih.gov/pmc/articles/PMC2693783/ (Accessed: 25 September 2019).

Edwards, M. (2017) *The Barker Hypothesis*. In: Preedy V., Patel V. (eds) Handbook of Famine, Starvation, and Nutrient Deprivation, pp.1–21. Springer, Cham. https://doi.org/10.1007/978-3-319-40007-5_71-1.

Elder, G.H., Johnson, M.K., Crosnoe, R. (2003). The Emergence and Development of Life Course Theory. In: Mortimer, J.T., Shanahan, M.J. (eds) Handbook of the Life Course. Handbooks of Sociology and Social Research. Springer, Boston, MA. https://doi.org/10.1007/978-0-306-48247-2_1.

Gergen, K.J. (1985) 'The social constructionist movement in modern psychology.' *American Psychologist*, 40(3), pp. 266–275.Available at:

https://www.swarthmore.edu/sites/default/files/assets/documents/kenneth-gergen/Social_Constructionist_Movement.pdf (Accessed: 3 January 2021).

Ghosh, G., Khan, H.T.A. and Vohra, S. (2021) 'The impact of obesity on health and social care needs among older adults (50+) in England.' PhD Thesis, The University of West London. Available at:https://repository.uwl.ac.uk/id/eprint/8510/1/Ghosh%20%20PhD%20Thesis%20(Final)%20Oct%2021.pdf. **(**Accessed: 27 November 2022).

Goering S. (2015) 'Rethinking disability: the social model of disability and chronic disease.' *Current reviews in musculoskeletal medicine*, 8(2), pp.134–138. https://doi.org/10.1007/s12178-015-9273-z.

GOV. UK (2005). *Mental Capacity Act 2005*. Legislation.gov.uk. Available at: https://www.legislation.gov.uk/ukpga/2005/9/contents. (Accessed: 25 January 2021).

Haberkern, F. K., Schmid, T., Neuberger, F. and Grignon, M. (2012) *The role of the elderly as providers and recipients of care.*: OECD Publishing. Publishing, Paris,189–257.

Han, T.S., Tajar, A. and Lean, M.E.J. (2011) 'Obesity and weight management in the elderly', *British Medical Bulletin*, 97, pp. 169–196. doi: 10.1093/bmb/ldr002.

Health Research Authority (2018) *The Integrated Research Application System.* version 5.8.0. Available at: https://www.myresearchproject.org.uk/ (Accessed: 28 April 2018).

Holstein, J.A. and Miller, G. (2007) *Reconsidering social constructionism: debates in social problems theory.* New Brunswick: Aldine transaction, NJ.

Holloway, I. and Freshwater, D. (2007) *Narrative research in nursing*. Oxford, Uk; Malden, Ma: Blackwell Pub. Available at: https://www.wiley.com/en-ec/Narrative+Research+in+Nursing-p-9781405114080. **(**Accessed: 12 December 2020).

Hosking, D.L. (2008) *Critical Disability Theory*. A paper presented at the 4th Biennial Disability Studies Conference, Lancaster University, UK. Available at: https://www.lancaster.ac.uk/fass/events/disabilityconference_archive/2008/papers/hosking2008.pdf. **(**Accessed: 16 November 2018).

Jacob, C.M., Baird, J., Baker, M., Cooper, C. and Hanson, M. (2017) *The importance of a life-course approach to health: Chronic disease risk from preconception through adolescence and adulthood*. World Health Organization. Available at: https://www.who.int/life-course/publications/importance-of-life-course-approach-to-health/en/ (Accessed: 16 November 2018).

Jung, Franziska U. and Luck-Sikorski, C. (2019) 'Overweight and Lonely? A Representative Study on Loneliness in Obese People and Its Determinants'. *Obesity Facts*, 12(4), pp.440–447. doi: 10.1159/000500095.

Kettner, P. M., Moroney, R. M. and Martin, L. L. (1990) *Designing and managing programs: An effectiveness-based approach*. Sage Publications. Newbury Park, CA.

LaMorte, W.W. (2019) *Behavioral Change Models: The Social Cognitive Theory*. Boston University School of Public Health. Available at: http://sphweb.bumc.bu.edu/otlt/MPH-Modules/SB/BehavioralChangeTheories/BehavioralChangeTheories5.html. (Accessed: 17 July 2020).

Leal Neto, J.D.S., Barbosa, A.R. and Meneghini, V. (2016) ‘Diseases and chronic health conditions, multimorbidity and body mass index in older adults.’ *Brazilian Journal of Kinanthropometry and Human Performance,* 18(5), pp.509-519. doi:10.5007/1980-0037.2016v18n5p509.

Lincoln, Y. S. and Guba, E. G. (1985) *Naturalistic inquiry*. Beverly Hills, CA: Sage.

Local Government Association (2020) *Social care and obesity*. Available at: https://local.gov.uk/publications/social-care-and-obesity (Accessed: 20 February 2021).

Mccartney, G. (2017) *Reducing health inequalities: insights from theory and practice*. NHS Health Scotland. Available at: https://www.scotpho.org.uk/media/1723/phins-2017-4-gerry-mccartney.pdf. (Accessed: 29 October 2018).

National Health Service (NHS) Digital (2020) *Statistics on Obesity, Physical Activity and Diet, England, 2020, Part 3: Adult overweight and obesity*. Available at: https://digital.nhs.uk/data-and-information/publications/statistical/statistics-on-obesity-physical-activity-and-diet/england-2020/part-3-adult-obesity-copy. **(**Accessed: 12 July 2020).

National Health Service (NHS) England (2017) *Unmet need in healthcare: Summary of a roundtable held at the Academy of Medical Sciences on 31 July 2017, held with support from the British Academy and NHS England*. The Academy of Medical Sciences. Available at: https://acmedsci.ac.uk/file-download/430378. (Accessed: 23 January 2021).

Ng, M., Fleming, T., Robinson, M., Thomson, B., Graetz, N., Margono, C., Mullany, E. C., Biryukov, S., Abbafati, C., Abera, S. F., Abraham, J. P. and Abu-Rmeileh, N. M. et al. (2014) ‘Global, regional, and national prevalence of overweight and obesity in children and adults during 1980-2013: a systematic analysis for the Global Burden of Disease Study 2013.’ *Lancet* (London, England), 384(9945), pp.766–781. https://doi.org/10.1016/S0140-6736(14)60460-8.

Nizalova, O., Gousia, K. and Forder, J.E. (2018) *Body mass, physical activity and future long-term care use*. QORU working paper, University of Kent. Available at: https://kar.kent.ac.uk/77852/ (Accessed: 20 August 2020).

Office for National Statistics (ONS) (2013) *Adult Health in Great Britain*. Available at: https://www.ons.gov.uk/peoplepopulationandcommunity/healthandsocialcare/healthandlifeexpectancies/compendium/opinionsandlifestylesurvey/2015-03 19/adulthealthingreatbritain2013. **(**Accessed: 6 March 2019).

Patton, M.Q. (2002) *Qualitative Research & Evaluation Methods*. SAGE. Available at: https://www.google.co.uk/books/edition/Qualitative_Research_Evaluation_Methods/FjBw2oi8El4C?hl=en&gbpv=1&dq=Patton+MQ.+2002.+Qualitative+research+%26+evaluation+methods [Accessed: 24 January 2021].

Rashbrook, E. (2019) *Health Matters: Prevention - A Life Course Approach - Public health matters*. Public Health England. Available at: https://publichealthmatters.blog.gov.uk/2019/05/23/health-matters-prevention-a-life-course-approach/. (Accessed: 17 January 2020).

Rössner, S. (2001) ‘Obesity in the elderly – a future matter of concern?’, *The International Association for the Study of Obesity, Obesity reviews*, 2(3), pp. 183–188. https://doi.org/10.1046/j.1467-789x.2001.00034.x.

Sach, T.H., Barton, G.R., Doherty, M., Muir, K.R., Jenkinson, C. and Avery, A.J. (2006) 'The relationship between body mass index and health-related quality of life: comparing the EQ-5D, EuroQol VAS and SF-6D'. *International Journal of Obesity*, 31(1), pp.189–196. doi: 10.1038/sj.ijo.0803365.

Shankar, A., Rafnsson, S.B. and Steptoe, A. (2015) 'Longitudinal associations between social connections and subjective wellbeing in the English Longitudinal Study of Ageing.' *Psychology & Health*, 30(6), pp.686–698. doi: 10.1080/08870446.2014.979823.

Sørbye, L.W., Schroll, M., Finne-Soveri, H. and Jónnson, P.V. et al. (2007) 'Home care needs of extremely obese elderly European women'. *Post Reproductive Health*. https://doi.org/10.1258/175404507780796406.

The United Kingdom Employment Appeal Tribunal database (2013) *Appeal No. UKEAT/0097/12/KN*. Walker v Sita Information Networking Computing Ltd UKEAT /0097/12/KN. Available at: https://www.employmentcasesupdate.co.uk/site.aspx?i=ed15996. **(**Accessed: 20 September 2018).

Thompson, J., Wittenberg, R., Henderson, C. and Darton, R. (2014) *Social care: need for and receipt of help*, (Chapter 2). Health Survey for England 2013. Available at: https://files.digital.nhs.uk/publicationimport/pub16xxx/pub16076/hse2013-ch2-soc-care.pdf (Accessed: 18 August 2018).

Tomlinson, D. J., Erskine, R. M., Morse, C. I., Winwood, K. and Onambélé-Pearson, G. (2016) 'The impact of obesity on skeletal muscle strength and structure through adolescence to old age.' *Biogerontology*, 17(3), pp.467–483. https://doi.org/10.1007/s10522-015-9626-4.

Trull, T.J., Vergés, A., Wood, P.K., Jahng, S. and Sher, K.J. (2012) 'The structure of Diagnostic and Statistical Manual of Mental Disorders (4th edition, text revision) personality disorder symptoms in a large national sample'. *Personality Disorders*, 3(4), pp.355–369. doi: 10.1037/a0027766.

Vlachantoni, A., Shaw, R. J., Evandrou, M. and Falkingham, J. (2015) 'The determinants of receiving social care in later life in England'. *Ageing and Society*, 35(2), pp. 321–345. https://doi.org/10.1017/S0144686X1300072X.

Vlachantoni, A., Shaw, R., Willis, R., Evandrou, M., Falkingham, J. and Luff, R. (2011) 'Measuring unmet need for social care amongst older people.' *Population Trends*, 145(1), pp.60–76. doi: 10.1057/pt.2011.17.

Wharton, S., Lau, D.C.W., Vallis, M., Sharma, A.M., Biertho, L., Campbell-Scherer, D., Adamo, K. and Alberga, A. et al. (2020) 'Obesity in adults: a clinical practice guideline.' *Canadian Medical Association Journal*, 192(31), pp. E875–E891. doi: https://doi.org/10.1503/cmaj.191707.

World Health Organization (WHO) (2020) *Body mass index - BMI*. Available at: https://www.euro.who.int/en/health-topics/disease-prevention/nutrition/a-healthy-lifestyle/body-mass-index-bmi. (Accessed: 5 July 2020).

Wood S. (2008) 'Setting Up a Minor Illness Clinic', *Practice Nurse*, 36(5), pp. 21-24. Available at: https://www.researchgate.net/publication/287542410_Setting_up_a_minor_illness_clinic. (Accessed: 12 April 2018).

 Click here to access/download;Figure;Figure 1.png

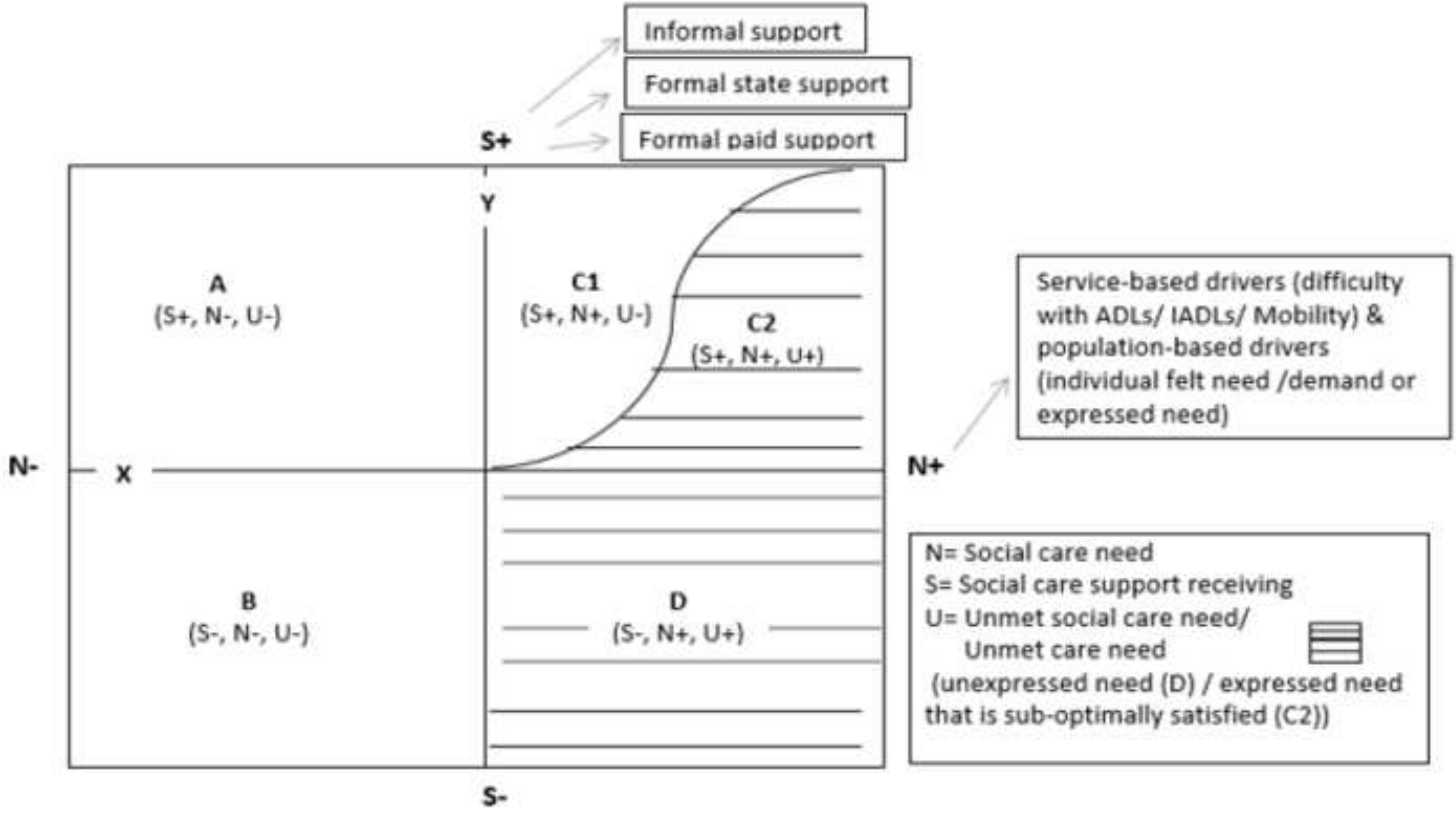

Click here to access/download
**Supporting Information**
Acknowledgement.docx

Interview Consent form

Click here to access/download
**Supporting Information**
Consent Form.docx

Click here to access/download
**Supporting Information**
Data Management and Storage Statement.docx

Click here to access/download
**Supporting Information**
GP Information Sheet.docx

Click here to access/download
**Supporting Information**
Participants information sheet.docx

Click here to access/download
**Supporting Information**
Semi-structured Questionnaires for Qualitative Interviews .docx

Dear editor,

I am resubmitting my revised manuscript as recommended by the academic editor, Maria Berghs, on 27/03/2024. The feedback I received was:

“Specifically: The English was not up to academic standards and the paper was difficult to read in places. You might want to consider proofreading and then resubmitting.”

I have included a tracked changes document of the manuscript to show the amendments made in response to the feedback. Please let me know if further clarification or details of the amendments are needed. I look forward to receiving positive feedback and guidance for publishing these important research findings.

Kind regards,

Dr Gargi Ghosh
