# Supplementary material for: A qualitative study to examine hidden care burden for older adults with overweight and obesity in England
Source: PLoS One. 2025 Mar 19;20(3):e0320253. doi: 10.1371/journal.pone.0320253 (PMC11922259; doi:10.1371/journal.pone.0320253)
Supplement: S3 File — (DOCX) [file pone.0320253.s003.docx]

Ethical Approval Letters

A. College of Nursing, Midwifery and Healthcare (CNMH) Decision Letter

Health Research Authority (HRA) and Health and Care Research Wales (HCRW) Approval Letter

| Professor Hafiz Khan  The Graduate School  University of West London  St Mary’s Road, Ealing, London  W5 5RF | Email: hra.approval@nhs.net HCRW.approvals@wales.nhs.uk |
| --- | --- |

20 December 2019

Dear Professor Khan

**HRA and Health and Care**

**Research Wales (HCRW)**

**Approval Letter**

**Research Wales (HCRW)**	**Approval Letter**

| **Study title:** | **The impact of obesity on health and social care needs among older adults (50+) in England** |
| --- | --- |
| **IRAS project ID:** | **253586** |
| **Protocol number:** | **21374279** |
| **REC reference:** | **19/LO/1093** |
| **Sponsor** | **University Of West London** |

I am pleased to confirm that **HRA and Health and Care Research Wales (HCRW) Approval** was given for the above referenced study, on the basis described in the application form, protocol, supporting documentation and any clarifications received. You should not expect to receive anything further relating to this application.

Please now work with participating NHS organisations to confirm capacity and capability, in line with the instructions provided in the “Information to support study set up” section towards the end of this letter.

**How should I work with participating NHS/HSC organisations in Northern Ireland and Scotland?**

HRA and HCRW Approval does not apply to NHS/HSC organisations within Northern Ireland and Scotland.

If you indicated in your IRAS form that you do have participating organisations in either of these devolved administrations, the final document set and the study wide governance report (including this letter) were sent to the coordinating centre of each participating nation. The relevant national coordinating function/s will contact you as appropriate.

Please see IRAS Help for information on working with NHS/HSC organisations in Northern Ireland and Scotland.

**How should I work with participating non-NHS organisations?**

HRA and HCRW Approval does not apply to non-NHS organisations. You should work with your non-NHS organisations to obtain local agreement in accordance with their procedures.

**What are my notification responsibilities during the study?**

The standard conditions document “*After Ethical Review – guidance for sponsors and investigators*”, issued with your REC favourable opinion, gives detailed guidance on reporting expectations for studies, including:

- Registration of research
- Notifying amendments
- Notifying the end of the study

The HRA website also provides guidance on these topics and is updated in the light of changes in reporting expectations or procedures.

**Who should I contact for further information?**

Please do not hesitate to contact me for assistance with this application. My contact details are below.

Your IRAS project ID is **253586**. Please quote this on all correspondence.

Yours sincerely,

Rekha Keshvara

Approvals Manager

Email: hra.approval@nhs.net

*Copy to:	Professor Heather Loveday*	**List of Documents**

The final document set assessed and approved by HRA and HCRW Approval is listed below.

|  | *Document* | | | | *Version* | | *Date* | |  |  |  |  |
| --- | --- | --- | --- | --- | --- | --- | --- | --- | --- | --- | --- | --- |
|  | Confirmation of any other Regulatory Approvals (e.g., CAG) and all correspondence [CCGs approval] | | | | v0.1 | | 20 July 2018 | |  |  |  |  |
|  | Contract/Study Agreement template [Registration MPhil/PhD] | | | | v0.2 | | 22 May 2018 | |  |  |  |  |
|  | Covering letter on headed paper [Cover letter] | | | | V0.1 | | 10 May 2019 | |  |  |  |  |
|  | Covering letter on headed paper [Word] | | | | V 0.2 | | 03 October 2019 | |  |  |  |  |
|  | Evidence of Sponsor insurance or indemnity (non-NHS Sponsors only) [Insurance] | | | | v0.1 | | 31 July 2018 | |  |  |  |  |
|  | GP/consultant information sheets or letters [Word] | | | | v0.1 | | 22 May 2018 | |  |  |  |  |
|  | HRA Schedule of Events | | | | 2 | | 19 December 2019 | |  |  |  |  |
|  | IRAS Application Form [IRAS_Form_03062019] | | | |  | | 03 June 2019 | |  |  |  |  |
|  | IRAS Checklist XML [Checklist_03062019] | | | |  | | 03 June 2019 | |  |  |  |  |
|  | IRAS Checklist XML [Checklist_23102019] | | | |  | | 23 October 2019 | |  |  |  |  |
|  | Letter from sponsor [Sponsorship letter] | | | | v0.1 | | 11 October 2018 | |  |  |  |  |
|  | Organisation Information Document | | | |  | |  | |  |  |  |  |
|  | Other [IRAS ethical review revised table] | | | | V 0.1 | | 07 October 2019 | |  |  |  |  |
|  | Other [Recruitment Clarification] | | | |  | | 25 June 2019 | |  |  |  |  |
|  | Participant consent form [Word] | | | | V 0.2 | | 01 October 2019 | |  |  |  |  |
|  | Participant information sheet (PIS) [Word] | | | | V 0.2 | | 01 October 2019 | |  |  |  |  |
|  | Participant information sheet (PIS) | | | | 0.3 | | 04 December 2019 | |  |  |  |  |
|  | Referee's report or other scientific critique report [Word] | | | | V0.1 | | 29 July 2018 | |  |  |  |  |
|  | Research protocol or project proposal [Pdf] | | | | V0.1 | | 31 July 2018 | |  |  |  |  |
|  | Summary CV for Chief Investigator (CI) [Pdf] | | | | V0.1 | | 26 September 2018 | |  |  |  |  |
|  | Summary CV for student [Gargi's CV] | | | | V0.1 | | 02 October 2018 | |  |  |  |  |
|  | Summary CV for supervisor (student research) [Academic co-supervisor] | | | | V0.1 | | 27 September 2018 | |  |  |  |  |
|  | Summary CV for supervisor (student research) [Co-supervisor's CV] | | | | V 0.1 | | 07 October 2019 | |  |  |  |  |
|  | Summary, synopsis or diagram (flowchart) of protocol in non-technical language [Conceptual Framework] | | | | V0.1 | | 24 May 2018 | |  |  |  |  |
|  | Validated questionnaire [Word] | | | | V0.1 | | 22 May 2018 | |  |  |  |  |
|  | Validated questionnaire [Word] | | | | V 0.2 | | 01 October 2019 | |  |  |  |  |
|  | **Information to support study set up**  The below provides all parties with information to support the arranging and confirming of capacity and capability with participating NHS organisations in England and Wales. This is intended to be an accurate reflection of the study at the time of issue of this letter. | | | | | | | |  |  |  |  |
|  | | | | | | | | | | | **IRAS project ID** | **253586** |
| **Types of participating**  **NHS**  **organisation** | | **Expectations related to confirmation of capacity and capability** | **Agreement to be used** | **Funding arrangements** | | **Oversight expectations** | | **HR Good Practice Resource Pack expectations** | |  |  |  |
| There is only one participating  NHS  organisation therefore there is only one site type. | | Research activities should not commence at participating NHS organisations in England or Wales prior to their formal confirmation of capacity and capability to deliver the study. | An Organisation  Information  Document has submitted and the sponsor is not requesting and does not expect any other site agreement to be used. | No study funding will be provided to sites as per the  Organisation  Information Document. | | A Local Collaborator should be appointed at study sites. | | It is expected that the principles of the HR Good Practice Pack are followed for researchers working in primary care. Researchers are advised to follow the processes of the local  primary care management function | |  |  |  |

**Other information to aid study set-up and delivery**

*This details any other information that may be helpful to sponsors and participating NHS organisations in England and Wales in study set-up.*

The applicant has indicated that they do not intend to apply for inclusion on the NIHR CRN Portfolio.

Research Ethics Committee (REC) Decision Letter

**London - Southeast Research Ethics Committee**

Barlow House

3rd Floor

4 Minshull Street

Manchester

M1 3DZ

Telephone: 0207 1048191

**Please note: This is the favourable opinion of the REC only and does not allow you to start your study at NHS** **sites in England until you** **receive HRA Approval.**

11 November 2019

Professor Hafiz Khan

The Graduate School

University of West London

St Mary’s Road, Ealing, London

W5 5RF

Dear Professor Khan

| **Study title:** | **The impact of obesity on health and social care needs among older adults (50+) in England** |
| --- | --- |
| **REC reference:** | **19/LO/1093** |
| **Protocol number:** | **21374279** |
| **IRAS project ID:** | **253586** |

Thank you for your letter of 3^rd^ October 2019 responding to the Proportionate Review.

Sub-Committee’s request for changes to the documentation for the above study.

The revised documentation was reviewed and approved on behalf of the PR sub-committee.

**Confirmation of ethical opinion**

On behalf of the Committee, I am pleased to confirm a favourable ethical opinion for the above research on the basis described in the application form, protocol and supporting documentation as revised.

**Conditions of the favourable opinion**

The REC favourable opinion is subject to the following conditions being met prior to the start of the study.

Confirmation of Capacity and Capability (in England, Northern Ireland and Wales) or NHS management permission (in Scotland) should be sought from all NHS organisations involved in the study in accordance with NHS research governance arrangements. Each NHS organisation must confirm through the signing of agreements and/or other documents that it has given permission for the research to proceed (except where explicitly specified otherwise).

Guidance on applying for HRA and HCRW Approval (England and Wales)/ NHS permission for research is available in the Integrated Research Application System.

For non-NHS sites, site management permission should be obtained in accordance with the procedures of the relevant host organisation.

Sponsors are not required to notify the Committee of management permissions from host organisations.

Registration of Clinical Trials

It is a condition of the REC favourable opinion that **all clinical trials are registered** on a publicly accessible database. For this purpose, ‘clinical trials’ are defined as the first four project categories in IRAS project filter question 2. Registration is a legal requirement for clinical trials of investigational medicinal products (CTIMPs), except for phase I trials in healthy volunteers (these must still register as a condition of the REC favourable opinion).

Registration should take place as early as possible and within six weeks of recruiting the first research participant at the latest. Failure to register is a breach of these approval conditions, unless a deferral was agreed by or on behalf of the Research Ethics Committee (see here for more information on requesting a deferral:

https://www.hra.nhs.uk/planning-and-improving-research/research-planning/research-registratio n-research-project-identifiers/

As set out in the UK Policy Framework, research sponsors are responsible for making information about research publicly available before it starts e.g., by registering the research project on a publicly accessible register. Further guidance on registration is available at:

https://www.hra.nhs.uk/planning-and-improving-research/research-planning/transparency-respo nsibilities/

You should notify the REC of the registration details. We routinely audit applications for compliance with these conditions.

**It is the responsibility of the sponsor to ensure that all the conditions are complied with before the start of the study or its initiation at a particular site (as applicable).**

**After ethical review: Reporting requirements**

The attached document “After ethical review – guidance for researchers” gives detailed guidance on reporting requirements for studies with a favourable opinion, including:

- Notifying substantial amendments
- Adding new sites and investigators
- Notification of serious breaches of the protocol
- Progress and safety reports
- Notifying the end of the study, including early termination of the study
- Final report

The latest guidance on these topics can be found at https://www.hra.nhs.uk/approvals-amendments/managing-your-approval/.

**Ethical review of research sites**

The favourable opinion applies to all NHS/HSC sites taking part in the study, subject to management permission being obtained from the NHS/HSC R&D office prior to the start of the study (see “Conditions of the favourable opinion” above).

**Approved documents**

The documents reviewed and approved by the Committee are:

| *Document* | *Version* | *Date* |
| --- | --- | --- |
| Confirmation of any other Regulatory Approvals (e.g., CAG) and all correspondence [CCGs approval] | v0.1 | 20 July 2018 |
| Contract/Study Agreement template [Registration MPhil/PhD] | v0.2 | 22 May 2018 |
| Covering letter on headed paper [Cover letter] | V0.1 | 10 May 2019 |
| Covering letter on headed paper [Word] | V 0.2 | 03 October 2019 |
| Evidence of Sponsor insurance or indemnity (non-NHS Sponsors only) [Insurance] | v0.1 | 31 July 2018 |
| GP/consultant information sheets or letters [Word] | v0.1 | 22 May 2018 |
| IRAS Application Form [IRAS_Form_03062019] |  | 03 June 2019 |
| IRAS Checklist XML [Checklist_03062019] |  | 03 June 2019 |
| IRAS Checklist XML [Checklist_23102019] |  | 23 October 2019 |
| Letter from sponsor [Sponsorship letter] | v0.1 | 11 October 2018 |
| Other [Recruitment Clarification] |  | 25 June 2019 |
| Other [IRAS ethical review revised table] | V 0.1 | 07 October 2019 |
| Participant consent form [Word] | V 0.2 | 01 October 2019 |
| Participant information sheet (PIS) [Word] | V 0.2 | 01 October 2019 |
| Referee's report or other scientific critique report [Word] | V0.1 | 29 July 2018 |
| Research protocol or project proposal [Pdf] | V0.1 | 31 July 2018 |
| Summary CV for Chief Investigator (CI) [Pdf] | V0.1 | 26 September 2018 |
| Summary CV for student [Gargi's CV] | V0.1 | 02 October 2018 |
| Summary CV for supervisor (student research) [Academic co-supervisor] | V0.1 | 27 September 2018 |
| Summary CV for supervisor (student research) [Co-supervisor's CV] | V 0.1 | 07 October 2019 |
| Summary, synopsis or diagram (flowchart) of protocol in non-technical language [Conceptual Framework] | V0.1 | 24 May 2018 |
| Validated questionnaire [Word] | V0.1 | 22 May 2018 |
| Validated questionnaire [Word] | V 0.2 | 01 October 2019 |

**Statement of compliance**

The Committee is constituted in accordance with the Governance Arrangements for Research Ethics Committees and complies fully with the Standard Operating Procedures for Research Ethics Committees in the UK.

**User Feedback**

The Health Research Authority is continually striving to provide a high-quality service to all applicants and sponsors. You are invited to give your view of the service you have received and the application procedure. If you wish to make your views known, please use the feedback form available on the HRA website:

http://www.hra.nhs.uk/about-the-hra/governance/quality-assurance/

**HRA Learning**

We are pleased to welcome researchers and research staff to our HRA Learning Events and online learning opportunities– see details at:

https://www.hra.nhs.uk/planning-and-improving-research/learning/

| **19/LO/1093** |  |  | **Please quote this number on all correspondence** |
| --- | --- | --- | --- |

With the Committee’s best wishes for the success of this project.

Yours sincerely

*PP*

**Chair**

Email: nrescommittee.london-southeast@nhs.net

Enclosures:	“After ethical review – guidance for researchers”
