# Supplementary material for: A qualitative study to examine hidden care burden for older adults with overweight and obesity in England
Source: PLoS One. 2025 Mar 19;20(3):e0320253. doi: 10.1371/journal.pone.0320253 (PMC11922259; doi:10.1371/journal.pone.0320253)
Supplement: Attachment — Submitted filename: Response_to_Reviewers_auresp_2.docx [file pone.0320253.s010.docx]

| **Reviewer and editorial comments** | **Amendment details** | **The page number on the Manuscript (if any)** |
| --- | --- | --- |
| 1. Objective of the Study: It is written as quantitative using verb “to examine the association between …” Qualitative studies are not intended for examining the association rather focus on exploration and understanding the phenomena. Revise the study objective to better align with qualitative research language. | Amended the study objective as recommended | pp-2 & 5 |
| 2. Ethical Consideration: Simplify the ethical consideration section to retain essential details while reducing verbosity. | Amended as recommended | PP-7 |
| 3. Abstract: Provide a concise background that highlights the research gap in 1-2 line. | A concise background is provided highlighting the research gap | PP-2 |
| 4. Introduction: It is very lengthy and difficult to follow. Streamline the introduction by focusing on the key background, research gap, and objectives in a concise manner. | I have revised the introduction as recommended, reducing its length from four pages to two and a half pages. | pp-4, 5 & 6 |
| 5. Methods: All sections are overly detailed, resembling a thesis. Please revise to present the content concisely, particularly in the ethical aspect and underpinning theory sections. Condense sections while maintaining clarity.  Underpinning Theory: Limit to 1–2 paragraphs with only relevant points for this study, omitting extensive theoretical details. Discussion of underpinning theory should be condensed into a few sentences that directly tie to the study.  Eligibility: Combine bullet points into a paragraph  Try to avoid using terms " like quantitative study. | I have revised the methodology section, including the ethical aspects, theory, and eligibility, as recommended. The theory section is now two paragraphs long. For eligibility, I merged the bullet points into a single paragraph. Additionally, terms such as "measurement of variables" have been removed. | pp- 6, 7 & 10 |
| 6. Conclusion: Focus on summarizing the study's findings and implications in a concise manner, avoiding unrelated comparisons (line 4) like discussion. Avoid using terms like "outcomes" in the context of qualitative research. | I have revised the conclusion as recommended. | PP-33 & 34 |
| 7. Overall Manuscript: Throughout the manuscript, prioritize brevity by including only key information directly relevant to the study. Remove excessive elaboration (e.g., thesis-level details). Ensure a clear flow by presenting information systematically, avoiding redundancy. | I have thoroughly revised the manuscript as recommended, ensuring a logical flow by presenting information systematically and eliminating redundancy. |  |
