# Supplementary material for: A qualitative study to examine hidden care burden for older adults with overweight and obesity in England
Source: PLoS One. 2025 Mar 19;20(3):e0320253. doi: 10.1371/journal.pone.0320253 (PMC11922259; doi:10.1371/journal.pone.0320253)
Supplement: Attachment — Submitted filename: Letter to editor.docx [file pone.0320253.s008.docx]

Dear editor,

I am resubmitting my revised manuscript as recommended by the academic editor, Maria Berghs, on 27/03/2024. The feedback I received was:

“Specifically: The English was not up to academic standards and the paper was difficult to read in places. You might want to consider proofreading and then resubmitting.”

I have included a tracked changes document of the manuscript to show the amendments made in response to the feedback. Please let me know if further clarification or details of the amendments are needed. I look forward to receiving positive feedback and guidance for publishing these important research findings.

Kind regards,

Dr Gargi Ghosh
