# Supplementary material for: A qualitative study to examine hidden care burden for older adults with overweight and obesity in England
Source: PLoS One. 2025 Mar 19;20(3):e0320253. doi: 10.1371/journal.pone.0320253 (PMC11922259; doi:10.1371/journal.pone.0320253)
Supplement: Attachment — Submitted filename: Response to Reviewers.docx [file pone.0320253.s009.docx]

| **Reviewer and editorial comments** | **Amendment details** | **The page number on the Manuscript (if any)** |
| --- | --- | --- |
| 1. When submitting your revision, we need you to address these additional requirements.  Please ensure that your manuscript meets PLOS ONE's style requirements, including those for file naming. The PLOS ONE style templates can be found at  <https://journals.plos.org/plosone/s/file?id=wjVg/PLOSOne_formatting_sample_main_body.pdf> and  <https://journals.plos.org/plosone/s/file?id=ba62/PLOSOne_formatting_sample_title_authors_affiliations.pdf> | We have changed the heading and sub-heading styles, title page, in text citation & referencing style, fig & table requirements and supporting documents, as requested. |  |
| Competing Interests | Entered: The authors have declared that no  competing interests exist. | PP-1 |
| 2. We note that this data set consists of interview transcripts. Can you please confirm that all participants gave consent for interview transcript to be published?  **Ethics Statement**  Format for specific study types  Human Subject Research (involving human  participants and/or tissue)  Give the name of the institutional review  board or ethics committee that approved the  study  •  Include the approval number and/or a  statement indicating approval of this  research  •  Indicate the form of consent obtained  (written/oral) or the reason that consent was  not obtained (e.g. the data were analyzed  anonymously) | The ethical statement section under methods has been amended as recommended. Included the statement- “All participants provided written and signed consent prior to participation by completing the informed consent forms. The participants also consented to use their interview transcripts, with all identifiable details removed, for teaching, future research, and publication purposes. It is confirmed that the transcripts do not contain any identifying information. All participants in this study were assigned pseudonyms, with their interview data organised and anonymised using sequential case numbers for analysis” | PP-7 |
| Important: Stating ‘data available on request  from the author’ is not sufficient. If your data  are only available upon request, select ‘No’ for  the first question and explain your exceptional  situation in the text box. | Amended to 'No' – The original transcription cannot be submitted without participant consent; however, a version with all identifiable details removed has been provided within the manuscript in Table 2. |  |
| **Reviewer #1**  Methodology is extremely vigorous. | We have shortened the ‘Underpinning theory’ section. | pp- 8 & 9 |
| **Reviewer #2**  Write full spellings of abbreviations used in abstract and font issues, and repeated full stops (Mentioned with yellow highlight in the pdf file).  Need to short and avoid repeating the methodology section.  Page # 02: full spellings of NHS.  Page # 02: were all older participants (male or female or both) in this study, have British background or immigrant to British from different nationalities? Mentioned in Abstract. As later on mentioned in Table 1.  Table 2 can put all data in supplementary files. | Modified as requested.  Modified as requested.  Modified as requested.  Mentioned in abstract as requested with a sentence- “All participants in this study are either of British origin or immigrants to the UK from various nationalities.”  The author plans to retain Table 2 within the main body of the text, as both the results and discussions are directly tied to it. The author believes that without this table, it would be challenging for readers to fully grasp the study's findings and discussions. | PP-2 & 7  pp-2  pp-2  pp- 25 |
